# The two-component system WalKR provides an essential link between cell wall homeostasis and DNA replication in *Staphylococcus aureus*

**DOI:** 10.1128/mbio.02262-23

**Published:** 2023-10-16

**Authors:** Liam K. R. Sharkey, Romain Guerillot, Calum J. Walsh, Adrianna M. Turner, Jean Y. H. Lee, Stephanie L. Neville, Stephan Klatt, Sarah L. Baines, Sacha J. Pidot, Fernando J. Rossello, Torsten Seemann, Hamish E. G. McWilliam, Ellie Cho, Glen P. Carter, Benjamin P. Howden, Christopher A. McDevitt, Abderrahman Hachani, Timothy P. Stinear, Ian R. Monk

**Affiliations:** 1 Department of Microbiology and Immunology, Doherty Institute for Infection and Immunity, University of Melbourne, Melbourne, Victoria, Australia; 2 The Florey Institute of Neuroscience and Mental Health, Melbourne Dementia Research Centre, The University of Melbourne, Parkville, Victoria, Australia; 3 University of Melbourne Centre for Cancer Research, The University of Melbourne, Melbourne, Victoria, Australia; 4 Australian Regenerative Medicine Institute, Monash University, Melbourne, Victoria, Australia; 5 Department of Microbiology and Immunology, Centre for Pathogen Genomics, Doherty Institute for Infection and Immunity, University of Melbourne, Melbourne, Victoria, Australia; 6 Biological Optical Microscopy Platform, University of Melbourne, Melbourne, Victoria, Australia; St. Jude Children's Research Hospital, Memphis, Tennessee, USA

**Keywords:** *Staphylococcus aureus*, two-component regulatory systems, regulation of gene expression, essential genes, split luciferase, ChIP-seq, CRISPRi, RNA-seq, WalKR, WalR, DNA replication, DNA compaction

## Abstract

Among the 16 two-component systems in the opportunistic human pathogen *Staphylococcus aureus*, only WalKR is essential. Like the orthologous systems in other Bacillota, *S. aureus* WalKR controls autolysins involved in peptidoglycan remodeling and is therefore intimately involved in cell division. However, despite the importance of WalKR in *S. aureus*, the basis for its essentiality is not understood and the regulon is poorly defined. Here, we defined a consensus WalR DNA-binding motif and the direct WalKR regulon by using functional genomics, including chromatin immunoprecipitation sequencing, with a panel of isogenic *walKR* mutants that had a spectrum of altered activities. Consistent with prior findings, the direct regulon includes multiple autolysin genes. However, this work also revealed that WalR directly regulates at least five essential genes involved in lipoteichoic acid synthesis (*ltaS*): translation (*rplK*), DNA compaction (*hup*), initiation of DNA replication (*dnaA*, *hup*) and purine nucleotide metabolism (*prs*). Thus, WalKR in *S. aureus* serves as a polyfunctional regulator that contributes to fundamental control over critical cell processes by coordinately linking cell wall homeostasis with purine biosynthesis, protein biosynthesis, and DNA replication. Our findings further address the essentiality of this locus and highlight the importance of WalKR as a *bona fide* target for novel anti-staphylococcal therapeutics.

**IMPORTANCE:**

The opportunistic human pathogen *Staphylococcus aureus* uses an array of protein sensing systems called two-component systems (TCS) to sense environmental signals and adapt its physiology in response by regulating different genes. This sensory network is key to *S. aureus* versatility and success as a pathogen. Here, we reveal for the first time the full extent of the regulatory network of WalKR, the only staphylococcal TCS that is indispensable for survival under laboratory conditions. We found that WalKR is a master regulator of cell growth, coordinating the expression of genes from multiple, fundamental *S. aureus* cellular processes, including those involved in maintaining cell wall metabolism, protein biosynthesis, nucleotide metabolism, and the initiation of DNA replication.

## INTRODUCTION

*Staphylococcus aureus* is an opportunistic pathogen that causes a wide range of hospital- and community-acquired infections. Antibiotic-resistant strains, notably methicillin-resistant (MRSA) and vancomycin-intermediate (VISA) strains, are persistent problems, with last-line agents, such as vancomycin, linezolid, and daptomycin, commonly associated with treatment failure (1, 2). MRSA is a World Health Organization “priority antibiotic-resistant pathogen” for the research and development of new antibiotics. Mortality from serious *S. aureus* infection is high (20%–50% of bacteraemias) (3), and the socioeconomic burden of *S. aureus* disease is substantial (4).

*S. aureus* encodes 16 core genome two-component systems (TCSs) that allow the bacterium to sense and respond to a range of stimuli, providing regulatory flexibility in changing environments. Of these 16 TCSs, only WalKR is essential for cell viability under laboratory conditions (5–8). WalKR is a canonical TCS that is conserved amongst low-GC Gram-positive bacteria, comprised of a multi-domain transmembrane sensor histidine kinase (WalK) and DNA-binding response regulator (WalR) of the OmpR family (9, 10). Upon activation by signal(s), WalK auto-phosphorylates a conserved histidine residue (H385) and subsequently transfers the phosphoryl group to a conserved aspartate residue (D53) in WalR. Phosphorylated WalR binds to promoter regions of genes within the WalKR regulon, operating as either a transcriptional activator or a repressor.

WalKR is a master regulator of cell wall homeostasis through the control of a suite of autolysins (7, 11–13). Although the locus is highly conserved, several points of difference between bacterial genera suggest variation in the precise cellular function of the system and the associated mechanism(s) of the essentiality. WalKR is located within an operon of three to six genes that also includes a varying number of accessory factors. Two accessory genes, *yycH* and *yycI*, encode membrane-associated proteins that differ in their function across genera. In *S. aureus*, these proteins are activators of WalKR activity, while conversely in *Bacillus subtilis*, they are repressors (14, 15). A second key difference between the systems is how WalKR interacts with the division septum. In *B. subtilis*, WalK controls the expression of FtsZ (16), it is localized to the division septum in an FtsZ-dependent manner and interacts with proteins of the divisome (17). Consequently, WalKR essentiality in *B. subtilis* arises from the coordination of cell wall remodeling with cell division, in response to signaling via an extracellular Per Arnt Sim (PAS) domain (17–20). In *S. aureus*, WalK is also reported to localize to the division septum in growing cells (21). Despite this, there remains no evidence of interaction with proteins of the divisome and FtsZ has not been mapped to the staphylococcal WalKR regulon. WalKR-depleted *S. aureus* can be complemented with constitutively overexpressed autolysins *lytM* or *ssaA* to restore bacterial viability. However, the resultant cells have morphological defects and neither of these genes are themselves essential (6). The *B. subtilis* extracellular PAS domain of WalK senses peptidoglycan cleavage products generated by WalKR-regulated autolysins, leading to homeostatic control of cell wall remodeling (22). The signal sensed by the extracellular PAS domain in *S. aureus* is not known. However, WalK activity in staphylococci (and predicted in enterococci), but not in other Bacillota, is modulated through coordination of a divalent metal ion by an intracellular PAS domain (23), raising the possibility of differing roles in regulation beyond peptidoglycan biosynthesis in these genera.

The WalKR regulon in *S. aureus* has been determined by comparative transcriptomics by depleting WalKR (7, 12) or by the expression a constitutively active WalR phosphomimic amino acid substitution (D53E) (24, 25). These studies, coupled with motif searching using a WalR DNA-binding motif defined in *B. subtilis* (11), have built a partial map of the WalKR regulon that includes genes involved in cell wall homeostasis (7, 12) and virulence (24). Here, we applied a customized implementation of chromatin immunoprecipitation sequencing (ChIP-seq), to define a 17-bp *S*. *aureus* WalR consensus-binding motif and identify the regulation of several essential genes involved in lipoteichoic acid polymerization, ribosome biogenesis, purine nucleotide salvage/*de novo* synthesis, and DNA replication. These data connect the regulation of cell division with chromosomal replication in *S. aureus* for the first time and identify pathways outside the regulation of autolysins to explain the essentiality of WalKR.

## RESULTS

### Specific mutations in the *walKR* locus increase and decrease WalKR activity

We initially assembled a panel of isogenic mutants with altered WalK or WalR activity in the native context to understand regulation without under- or over-expression. These included two previously described “down” mutants with decreased WalKR activity: *S. aureus* NRS384 Δ*yycHI*, with the deletion of both WalKR auxiliary proteins (*yycH* and *yycI*) (14), and NRS384 WalR_T101A_, in which second-site PknB phosphorylation at residue T101 was abolished (26). We also selected two “up” mutants that have increased WalKR activity: NRS384 WalK_Y32C_ with a mutation in the first transmembrane domain [identified from a sectored Δ*yycHI* colony (27)] and NRS384 WalK_T389A_ that is predicted to prevent the dephosphorylation of WalR (28).

**Fig 1:**
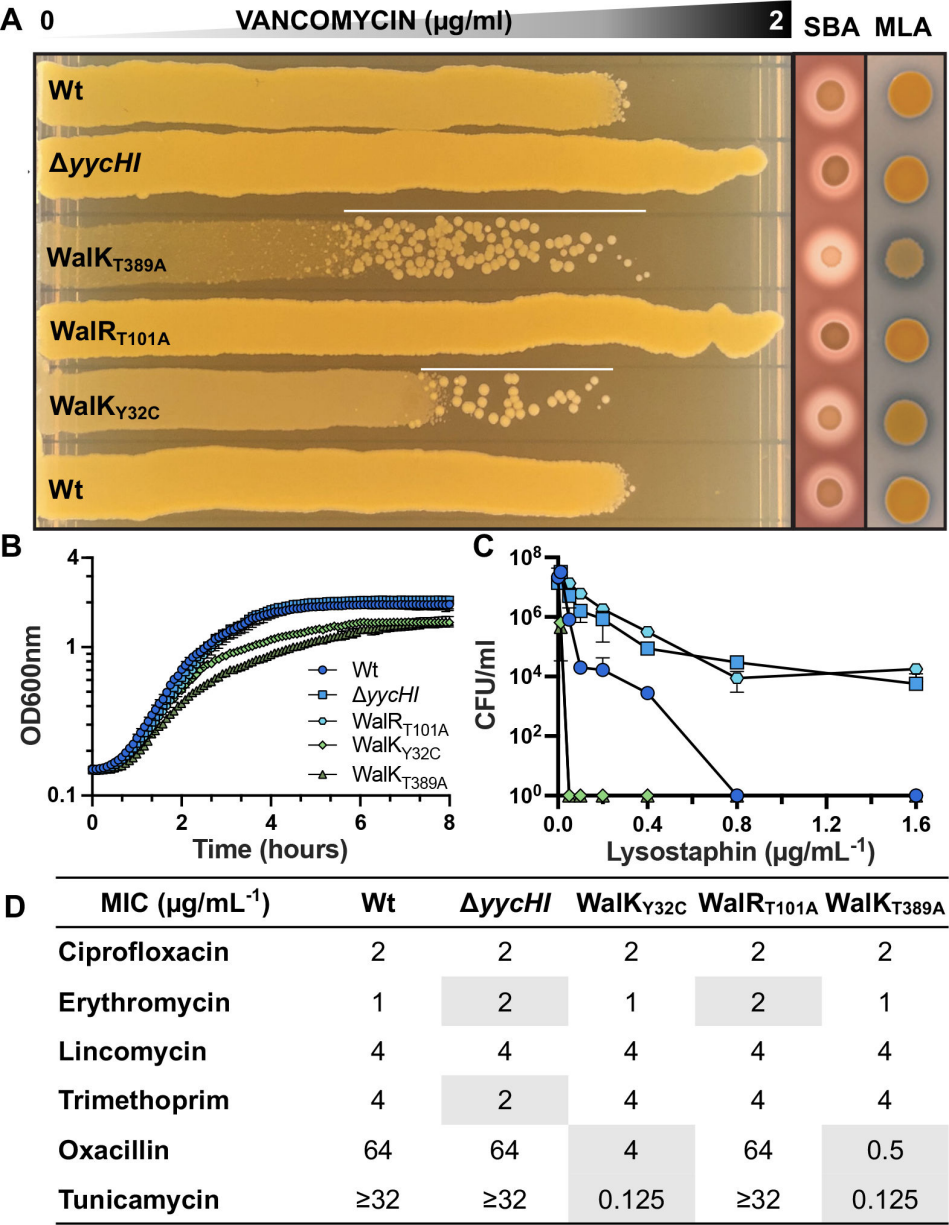
Phenotypic characterization of WalKR mutant strains. (**A**) Vancomycin gradient assay (VGA), sheep blood agar (SBA), and *Micrococcus luteus* agar (MLA) for the comparison of vancomycin susceptibility, hemolysis, and Atl-mediated autolytic activity, respectively. White line on VGA plates highlights suppressor mutants that arise from exposure to vancomycin in the “up” mutants. (**B**) Growth curves, (**C**) susceptibility to lysostaphin after a 90-min exposure, and (**D**) minimal inhibitory concentration of antibiotics against the different WalKR mutant strains, with differences to NRS384 wild type (Wt) highlighted.

We then confirmed that each mutant exhibited the expected phenotypes with “up” mutations resulting in an increased susceptibility to vancomycin and lysostaphin, increased hemolysis, reduced growth rate, and increased autolysis with the opposite true for “down” mutants (Fig. 1A through C) (12, 24, 27). Of the two “up” mutants, WalK_T389A_ showed the most prominent differences, suggesting the higher level of activation (Fig. 1A through C). Mutational activation of WalKR caused a striking increase in susceptibility to oxacillin (16- or 128-fold increase) and tunicamycin (>256-fold increase). Conversely, mutational dampening of WalKR activity caused a small but reproducible (twofold) decrease in erythromycin susceptibility, but we did not observe erythromycin or lincomycin hypersensitivity upon WalKR modulation as has previously been reported (5, 29). Susceptibility to compounds targeting other cellular pathways remained unchanged (Fig. 1D).

**Fig 2:**
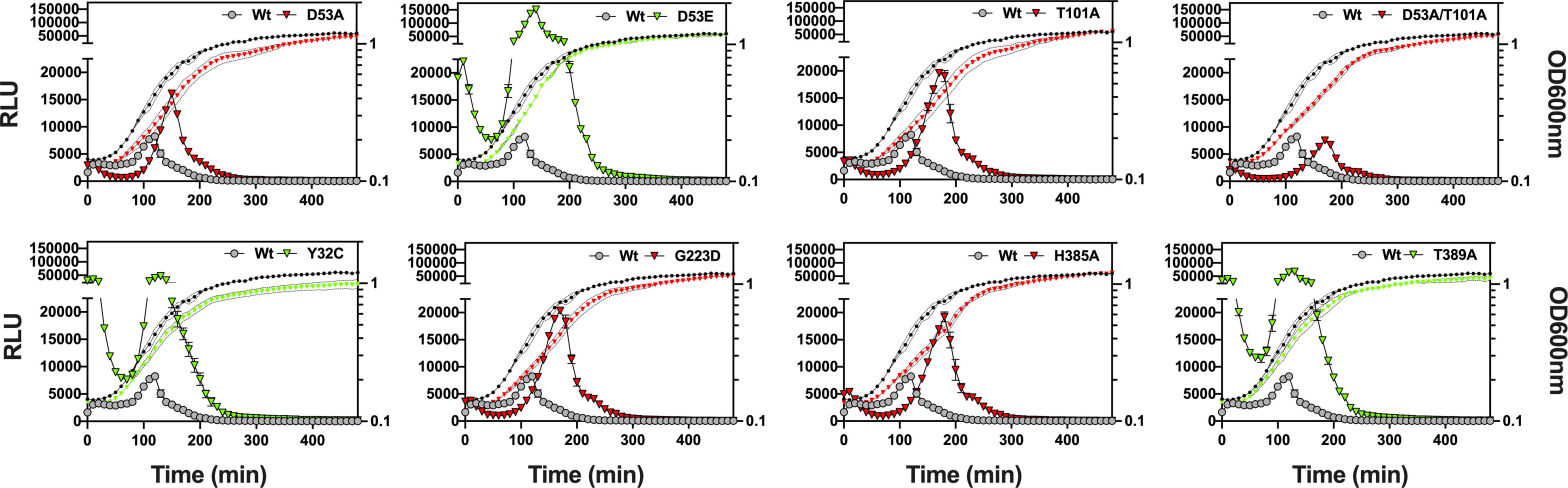
Kinetic interaction of WalR with WalK in *S. aureus*. Analysis of the interaction of WalR-SmBIT with WalK-LgBIT and WalK/WalR mutant proteins throughout growth in NRS384 Wt. OD_600nm_: open black circle (WalR/WalK Wt) and open red triangle (“down” mutant) or open green triangle (“up” mutant). RLU: gray-filled circle (WalR/WalK Wt) and either red- (“down” mutation) or green- (“up” mutation) filled triangle. Results are the mean from three independent determinations, and the error bars show the standard deviation.

TCS phosphoryl transfer from the sensor histidine kinase to the cognate DNA-binding response regulator requires direct interaction (30). To measure these interactions, we constructed WalR-SmBIT and WalK-LgBIT split luciferase fusions in a *S. aureus* dual-plasmid system with native and mutant proteins, following cell density (OD_600nm_) and light emission (RLU) throughout *S. aureus* growth (31). Chromosomal C-terminal tagging of WalR-SmBIT and WalK-LgBIT at the native locus showed that the proteins tolerated the presence of either tag, with no growth defect detected (Fig. S1). We observed immediate interaction of WalK with WalR upon dilution in fresh lysogeny broth (LB) with the peak WalK/WalR interaction in the mid-exponential phase of growth and subsequent rapid decline of the interaction to undetectable levels (5 h) in the stationary phase. This pattern of interaction was enhanced in the “up” mutants throughout growth (including lag phase) for both WalK_Y32C_, WalK_T389A_, and the previously described WalR_D53E_ mutant (24, 25) compared with the native WalK/WalR interaction (Fig. 2). In contrast, the “down” mutants, which included WalR_D53A_ (cannot be phosphorylated by WalK), WalR_T101A_, WalK_D53A/T101A_, WalK_G223D_ (reduced autophosphorylation and transfer to WalR) (32), and WalK_H385A_ (cannot phosphorylate WalR D53), exhibited a consistent profile of reduced initial WalK/WalR interaction during lag phase in comparison to the native WalK/WalR alleles (Fig. 2). The kinetic profile of the WalR_T101A_ mutant mimicked the interaction profile of the WalK_G223D_ mutant with a 1.5-fold increase in the maximal level of interaction (compared with native WalK/WalR). This increase correlated with the same mid-log phase time point as seen in the native interaction (Fig. 2). “Down” mutants also yielded detectable interaction into the stationary phase. These interaction profiles combined with phenotypic profiling validated our panel of “up” and “down” mutants.

### WalKR activation causes a global change in gene expression

**Fig 3:**
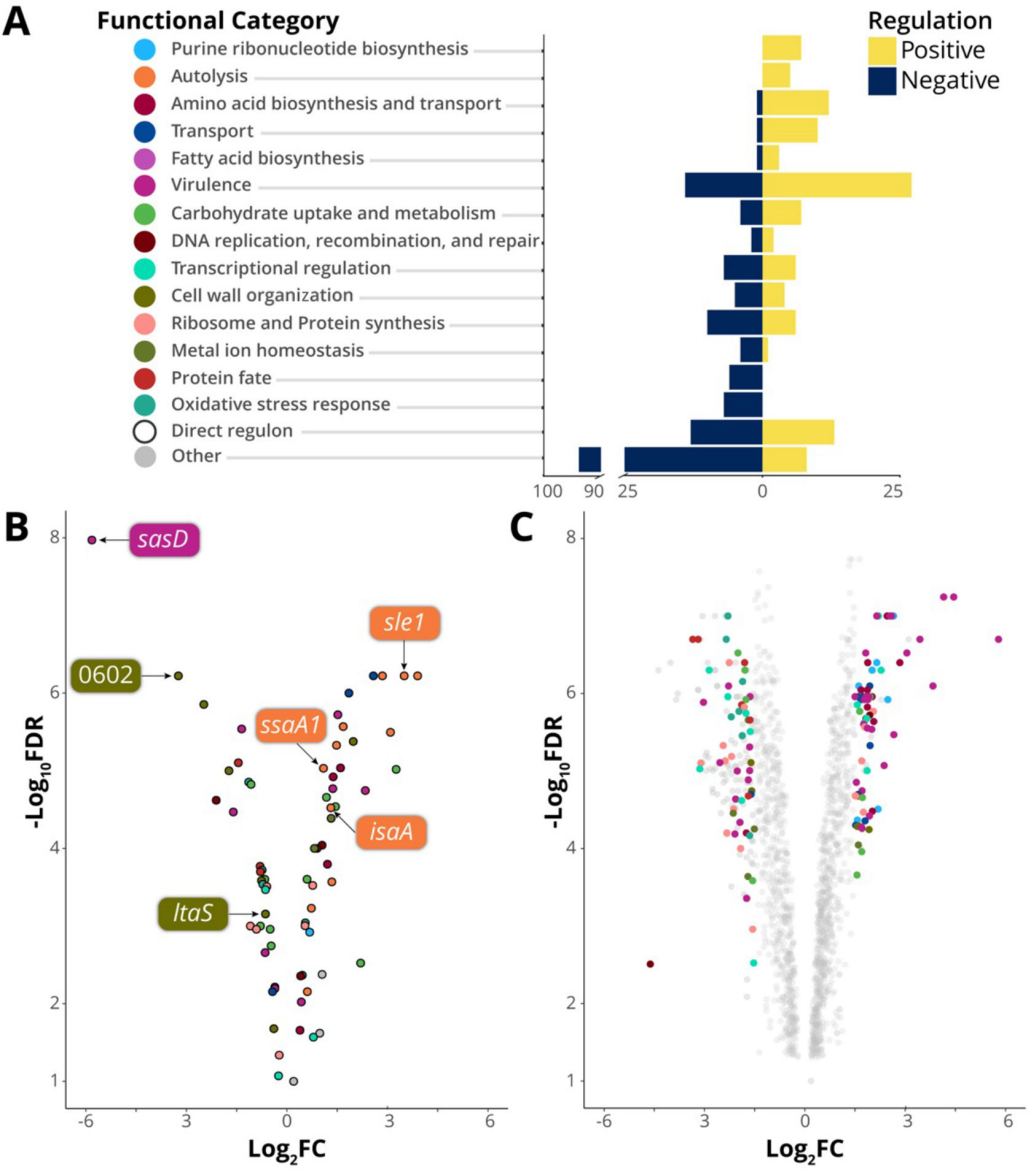
The direct and indirect WalKR regulon. (**A**) The number of genes in each category that is positively or negatively regulated upon WalK activation (FDR < 0.05, log_2_FC ≥ 1.5 or ≤−1.5). For clarity, categories where *n* < 2 are grouped with hypothetical genes in “other.” (**B**) Gene expression changes of the predicted direct WalKR regulon upon WalK activation (Wt vs WalK_T389A_). Genes with WalR-binding sites confirmed by ChIP-seq are highlighted (see Fig. 4D; Table S1). (**C**) Global change in transcriptome upon mutational activation of WalK (Wt vs WalK_T389A_); members of gene sets undergoing a log_2_FC ≥ 1.5 or ≤−1.5 are highlighted. *y*-axis labels in panel A serve as the legend for panels B and C.

To ascertain the impact of WalKR activation on *S. aureus* gene expression, we compared the transcriptome of “up” mutant WalK_T389A_ to the NRS384 strain. Activation of WalKR was associated with a global change in gene expression with 1,117 genes significantly regulated [false discovery rate (FDR) < 0.05, log_2_ fold change (FC) ≥ 0.585], 551 of which were increased in expression and 564 decreased (Fig. 3C). Genes with increased expression (≥1.5 log_2_FC, FDR < 0.05) included those encoding autolysins, virulence factors, membrane transporters, and proteins of the amino acid, purine, and fatty acid biosynthesis pathways (Fig. 3A; Table S1). Genes with decreased expression (log_2_FC ≤ −1.5, FDR < 0.05) were primarily involved in oxidative stress response, metal ion homeostasis, and protein fate (Fig. 3A; Table S1), whereas the expression of genes encoding ribosomal proteins, transcription factors, and proteins involved in carbohydrate metabolism, DNA maintenance, and cell wall organization had increased and reduced expression (FDR < 0.05, log_2_FC ≥ 1.5 or ≤−1.5) (Fig. 3A; Table S1).

Explanations for two of our observed WalKR phenotypes can be inferred in the WalK_T389A_ RNA sequencing (RNA-seq) data. Firstly, increased hemolysis (Fig. 1A) in WalK_T389A_ mutants is explained by an increase in *hla* expression (+1.7-log_2_FC), encoding alpha-toxin, upon WalK activation. Secondly, enhanced zones of clearing surrounding WalK_T389A_ on heat killed *Micrococcus luteus* agar plates (Fig. 1A) arise solely due to the increased peptidoglycan degradation of the secreted processed autolysin Atl (33) (+2.85-log_2_FC).

### Defining an *in vivo* WalR regulon using ChIP-seq

**Fig 4:**
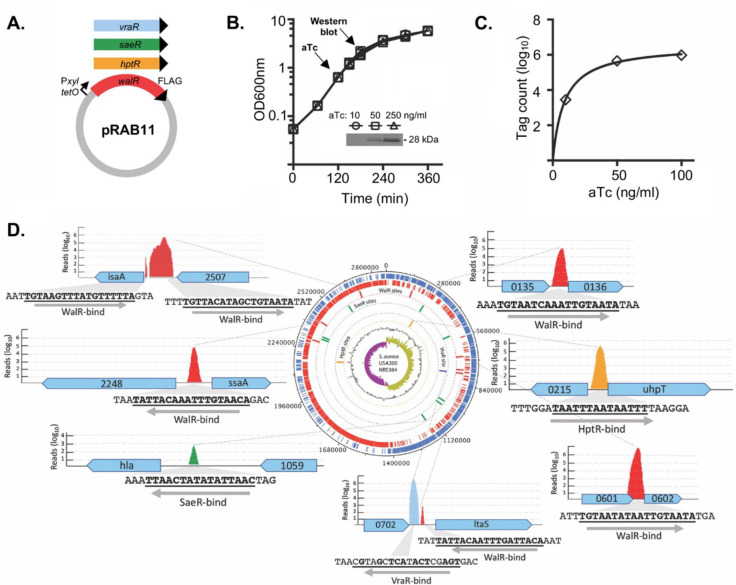
ChIP-seq analysis of *S. aureus* WalR chromosome-binding sites. (**A**) Four response regulators with a C-terminal FLAG-tag for immunoprecipitation after induction with anhydrotetracycline in NRS384Δ*spa*. (**B**) Dose-dependent production of WalR-FLAG (inset Western blot) with increasing concentrations of aTc. (**C**) Plot showing an increasing number of WalR ChIP-seq mapped reads (tag count) as the aTc concentration increases. (**D**) Summary of key ChIP-seq-binding sites across the *S. aureus* chromosome, showing binding motif and direction, including five WalR peaks (red), and single peaks for SaeR (green), VraR (blue), and HptR (yellow).

The direct regulon of WalR was then investigated using ChIP-seq. To permit immunoprecipitation, a 1xFLAG-tag was incorporated onto the C-terminus of WalR using a modified anhydrotetracycline (aTc)-inducible expression plasmid (34) and the *spa* gene, encoding protein A, was deleted from *S. aureus* NRS384. To validate functionally, the transcriptome of *S. aureus* NRS384 with chromosomally C-terminally FLAG tagged WalR (23) was compared with that of the wild-type strain. This strain had a gene expression profile that was like NRS384 Wt during the mid-log phase, as determined by RNA-seq. Subsequently, FLAG-tagged expression constructs were also made in pRAB11 for three other response regulators with known DNA-binding motifs, HptR, and SaeR and VraR (Fig. 4A) (35–37). Dose-dependent aTc induction was observed [induced at an optical density of 600 nm (OD_600nm_) of 0.6 for 1 h] for WalR (Fig. 4B). We examined the impact of increasing aTc concentrations on tag-counts (mapped sequence reads for ChIP-purified DNA) and selected an aTc concentration of 100 ng/mL^−1^ for subsequent ChIP-seq experiments (Fig. 4C). ChIP-seq was then conducted with each of the four constructs and the empty vector. Initial analysis of the resulting sequence reads revealed a high background of reads mapping across the entire *S. aureus* chromosome. To improve the signal-to-noise ratio for each of the four ChIP-seq experiments, we performed *in silico* subtraction of the read sets for the three non-target response regulators and the empty vector from the read set for the target response regulator (Fig. S2; Table S2). This revealed peaks anticipated for HptR and SaeR, upstream of *hpt* and *hla*, respectively (Fig. 4D; Table S1), and identified WalR-binding sites upstream of six genes that included autolysins SAUSA300_2249 (*ssaA*), SAUSA300_2506 (*isaA*), SAUSA300_0438 (*sle1*), cell wall cross-linked SAUSA300_0136 (*sasD*), hypothetical secreted SAUSA300_0602, and SAUSA300_0703 (*ltaS*) which encodes the essential lipoteichoic acid (LTA) synthase responsible for polymerizing glycerol-6-phosphate into LTA chains (38) (Fig. 4D; Table S1). All these genes had previously been identified as belonging to the WalKR regulon (7, 24). For VraR, we observed a ChIP-seq-binding site upstream of *ltaS* that was adjacent to the putative WalR-binding site (Fig. 4D).

Including the above six genes (seven peaks), a total of 22 WalR ChIP-seq peaks upstream of 21 genes were identified, with 12 set aside due to small peak size (7–20 nucleotides) and poor peak prediction scores. Two peaks were identified in 16S rRNA genes, but the use of ribosomal RNA depletion precluded interpretation of RNA-seq data for these loci. The peak upstream of SAUSA300_0681 was not pursued, as no candidate WalR-binding sites were identified (Table S1).

The high stringency required for the *in silico* subtraction approach may have eliminated peaks corresponding to lower affinity DNA-binding regions and thereby prevented identification of the complete direct WalR regulon. Consequently, we used ChIP-seq-defined WalR-binding regions in conjunction with previously validated WalR-binding sites (12, 24) to generate a 17-bp consensus *S. aureus* WalR-binding motif (5′-TGTHH[N]_6_WGTNDD-3′) (Fig. S3 A and B). This motif was the used to conduct an *in silico* search of the genome for potential WalR-binding sites. In total, 118 putative intergenic WalR-binding sites were identified within the NRS384 chromosome (Fig. S2B), of which 109 were within 500 bp of a predicted CDS transcriptional start site (Table S3).

### Positioning of WalR-binding site, not sequence or orientation, dictates the mode of regulation

To investigate whether the sequence of WalR motifs could determine the mode, or degree, of change in gene expression upon activation, WalR motif diversity was visualized as a maximum-likelihood phylogenetic tree and the tips labelled with gene expression changes upon WalK activation (Fig. S4). Functional groups of regulated genes were also mapped to examine whether specific motif signatures were linked to gene sets (Fig. S5). We did not observe clustering of positively or negatively regulated genes nor did branches of the tree correspond to specific gene functions (Fig. S4). Taken together, these analyses indicate that the sequence of WalR motifs does not dictate the mode of regulation nor is it linked to the functional class of the gene it controls.

Here, building on the framework used to analyze WalR in *B. subtilis* (13), we mapped the positions of *S. aureus* WalR-binding sites of the direct regulon in relation to predicted transcriptional start sites and promoters (39) (Table S4). The orientation of the WalR-binding site in relation to the downstream gene was not significantly associated with the magnitude (Student’s *t*-test, *P* = 0.52) or mode (*P* = 0.81) of expression change. However, the position of the WalR-binding site did dictate the mode of regulation; WalR binding upstream of the −35 element was significantly associated with positive regulation (*P* < 0.001), whereas WalR binding between, or downstream of, −35 and −10 elements was associated with negative regulation (*P* < 0.001) (Table S4; Fig. S4B). Thus, whether WalR activates or represses the expression of a gene is determined primarily by its position relative to the promoter rather than motif sequence.

### Functional validation of WalR directly regulated genes

**Fig 5:**
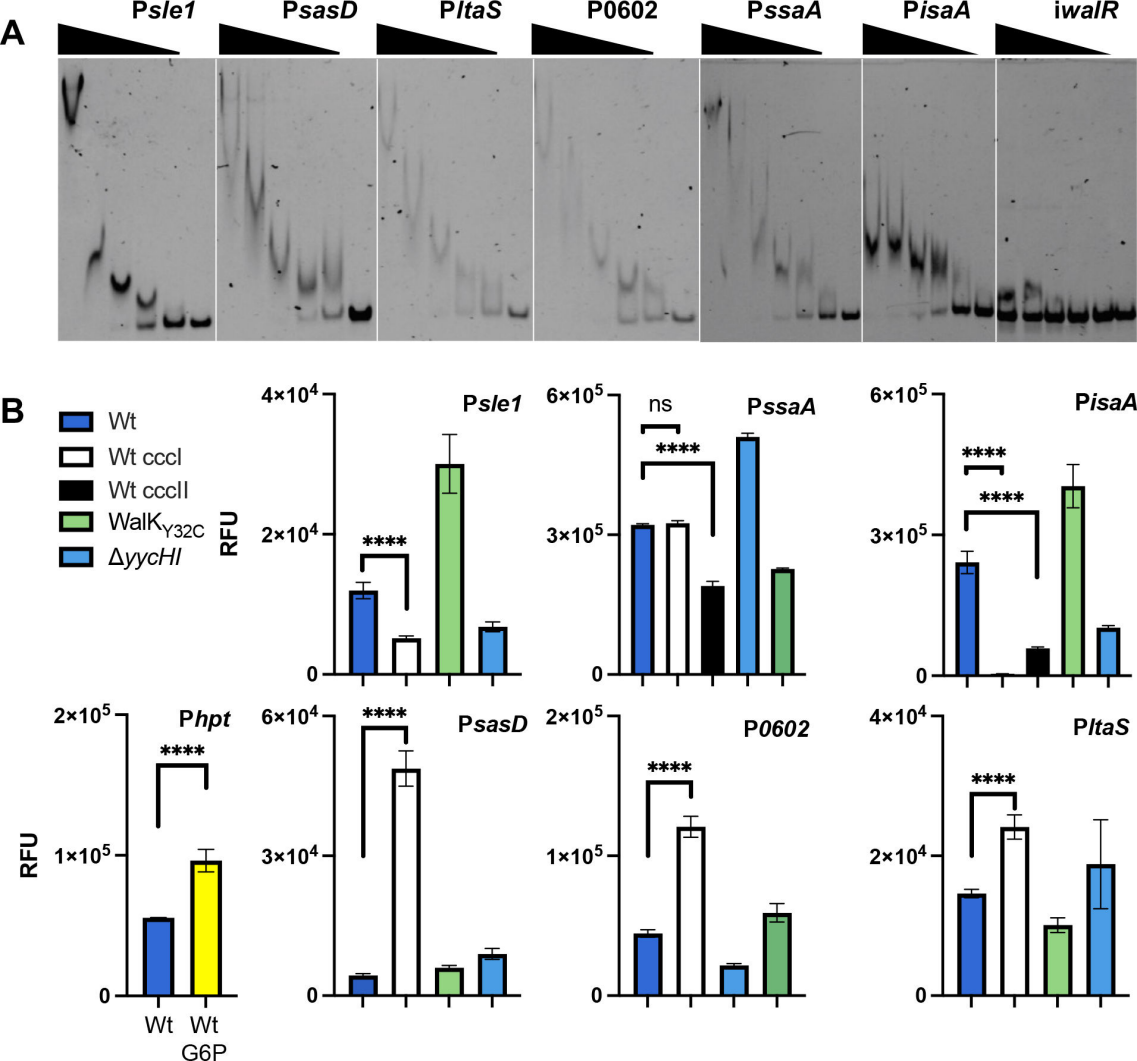
*In vitro* and in cellulo confirmation of WalR regulation. (**A**) Dose-dependent band shifts caused through specific WalR binding to the ChIP-seq-identified promoter regions. An intragenic region of the *walR* gene was used as a non-specific control (i*walR*). A total of five doubling dilutions (plus a no protein control—right lane) of WalR starting at 20 µM, for all except P*ssaA* where 40 µM was used. (**B**) Cumulative yellow fluorescent protein (YFP) fluorescence of *S. aureus* reporter strains from stationary phase cultures. The glucose-6-phosphate (G6P)-inducible hpt gene was used as a positive assay control through addition of 25 µM of G6P. Promoter activity was assessed in *S. aureus* NRS384, with or without an abrogating (ccc) mutation in the WalR-binding site. The native promoter construct was also analyzed in WalK_Y32C_ (“up” mutant) or Δ*yycHI* (“down” mutant) strains. Results are means from three independent experiments with the error bars showing the standard deviation of the mean. The significance of the ccc mutation was determined using an unpaired Student’s *t*-test; ns: not significant, <0.0001: **** versus the Wt promoter in NRS384.

We observed WalR binding to all six promoters identified by ChIP-seq using electrophoretic mobility shift assays (EMSAs) (Fig. 5A; Fig. S5 and S6). We also corroborated VraR binding to the *ltaS* promoter at the consensus VraR-binding motif (5′-TGA[N_1-3_]TCA-3′) (Fig. S7A) (35, 40). WalR had no affinity for this duplex (Fig. S7B). VraR did exhibit affinity for the WalR-binding site duplex; however, this was shown to be non-specific by competition assay (Fig. S7C).

We then assessed the impact of WalR binding on promoter activity in *S. aureus* (Fig. 5B). The promoter regions encompassing the WalR-binding motif and a mutated motif (first TGT changed to CCC) were transcriptionally fused with yellow fluorescent protein and transformed into *S. aureus* NRS384 Wt, “down” (Δ*yycHI*) and “up” (WalK_Y32C_) backgrounds. The WalK_Y32C_ strain was chosen rather than WalK_T389A_ as suppressor mutants arose in this background through genetic instability, which was not observed in WalK_Y32C_. As a positive control, we included the promoter for the *hpt* gene encoding the glucose-6-phosphate transporter which is responsive to the presence of glucose-6-phosphate (37) (Fig. 5B). Genes that were downregulated from RNA-seq (*sasD*, *ltaS*, and SAUSA300_0602), exhibited increased fluorescence upon abrogation of the WalR-binding motif, showed increased fluorescence in the “down” mutant (Δ*yycHI*), and decreased fluorescence in the “up” mutant (WalK_Y32C_). This is indicative of negative regulation by WalR (Fig. 5B). For upregulated genes, abrogation of the WalR-binding motif caused a reduction in fluorescence, with all showing increased activity in the WalK_Y32C_ mutant and decreased activity in the Δ*yycHI* mutant, indicative of positive WalR regulation (Fig. 5B). As the *isaA* and *ssaA* promoter regions contain two WalR-binding motifs, both were individually mutated. For *isaA*, the WalR-binding site closest to the transcriptional start site (TSS) caused the greatest decrease in promoter activity (27), whereas for *ssaA*, only the more distal binding site reduced expression, which corresponded to the single ChIP-seq peak for *ssaA* (Fig. 4D).

### Direct control of essential genes by WalKR

**Fig 6:**
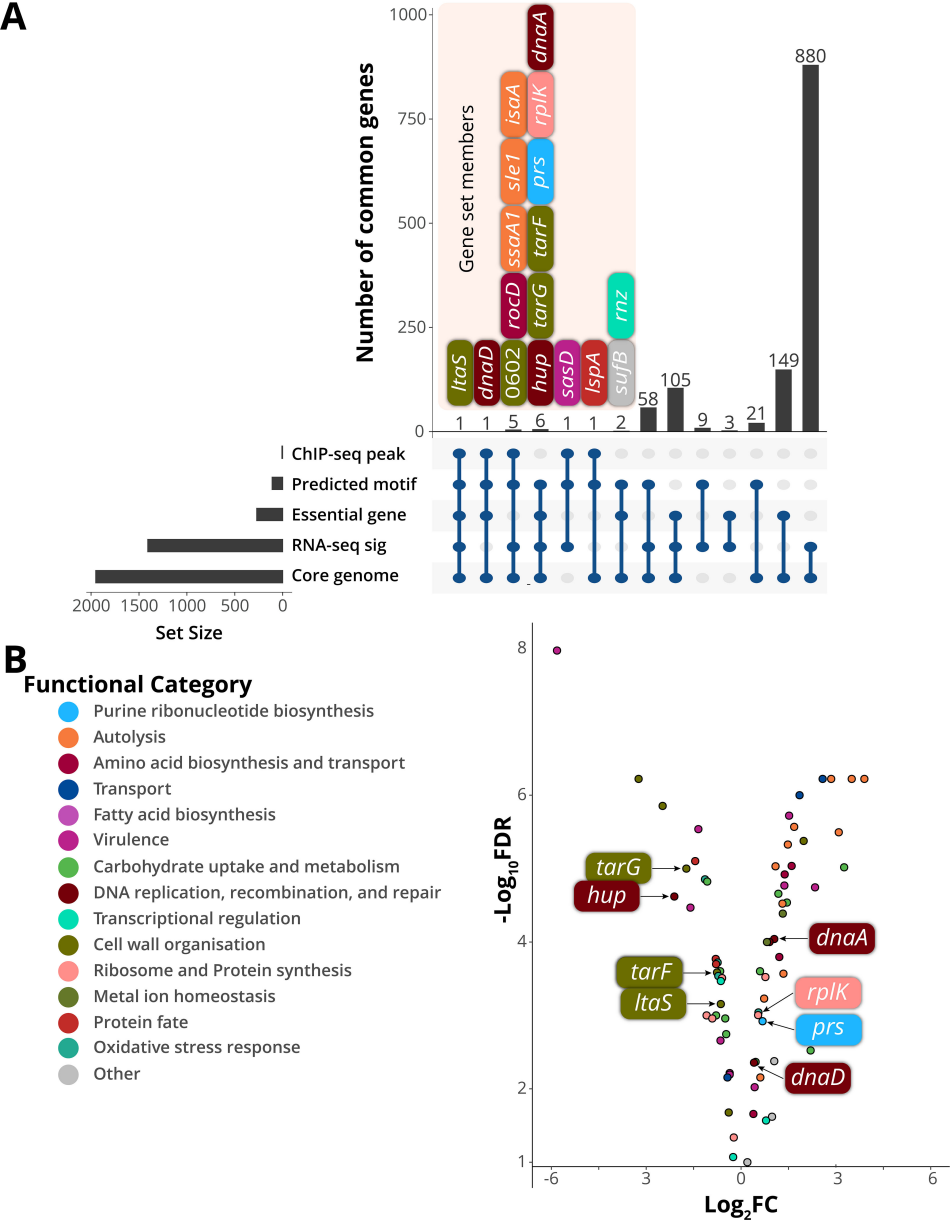
Analysis of putative WalR-controlled essential genes. (**A**) Intersections between essentiality analysis and -omics data displayed as an UpSet plot. Members of gene sets where *n* < 8 are shown. RNA-seq significance ((RNA-seq sig) FDR > 0.05, log2FC ≥ 0.585). (**B**) Gene expression changes of the predicted direct WalKR regulon upon WalK activation (Wt vs WalK_T389A_). Essential genes with predicted WalR-binding sites are highlighted.

To investigate WalKR essentiality in *S. aureus* and triage genes for further analysis based on their likely contribution to the essentiality phenotype, we analyzed intersecting data sets where genes fulfilled the following criteria: (i) contain a predicted upstream WalR-binding site, (ii) belong to the core *S. aureus* genome, (iii) essential for growth in rich media (41–43), and (iv) their expression is significantly changed upon WalK activation (Fig. 3A). We found that within the predicted direct WalR regulon, seven essential genes undergo a significant change in gene expression upon activation by WalKR (FDR > 0.05, log_2_FC ≥ 0.585) (Fig. 3A and B). These genes were *ltaS* (see above); *dnaA*, which encodes chromosomal replication initiator protein; *hup*, the sole DNA-binding protein HU; *prs*, which encodes a ribose-phosphate pyrophosphokinase involved in purine salvage and *de novo* synthesis; *rplK*, ribosomal protein L11 (50S subunit component); and *tagG* and *tarF*, which encode teichoic acid biosynthetic proteins (Fig. 6A and B). Additionally, three essential genes had a predicted upstream WalR-binding site but did not undergo a significant change in expression: *dnaD*, *rnz*, and *sufB*, encoding putative replication restart protein DnaD, ribonuclease Z, and FeS assembly protein SufB. Of these three essential genes, a ChIP-seq peak was identified upstream of *dnaD* (Fig. 6A).

**Fig 7:**
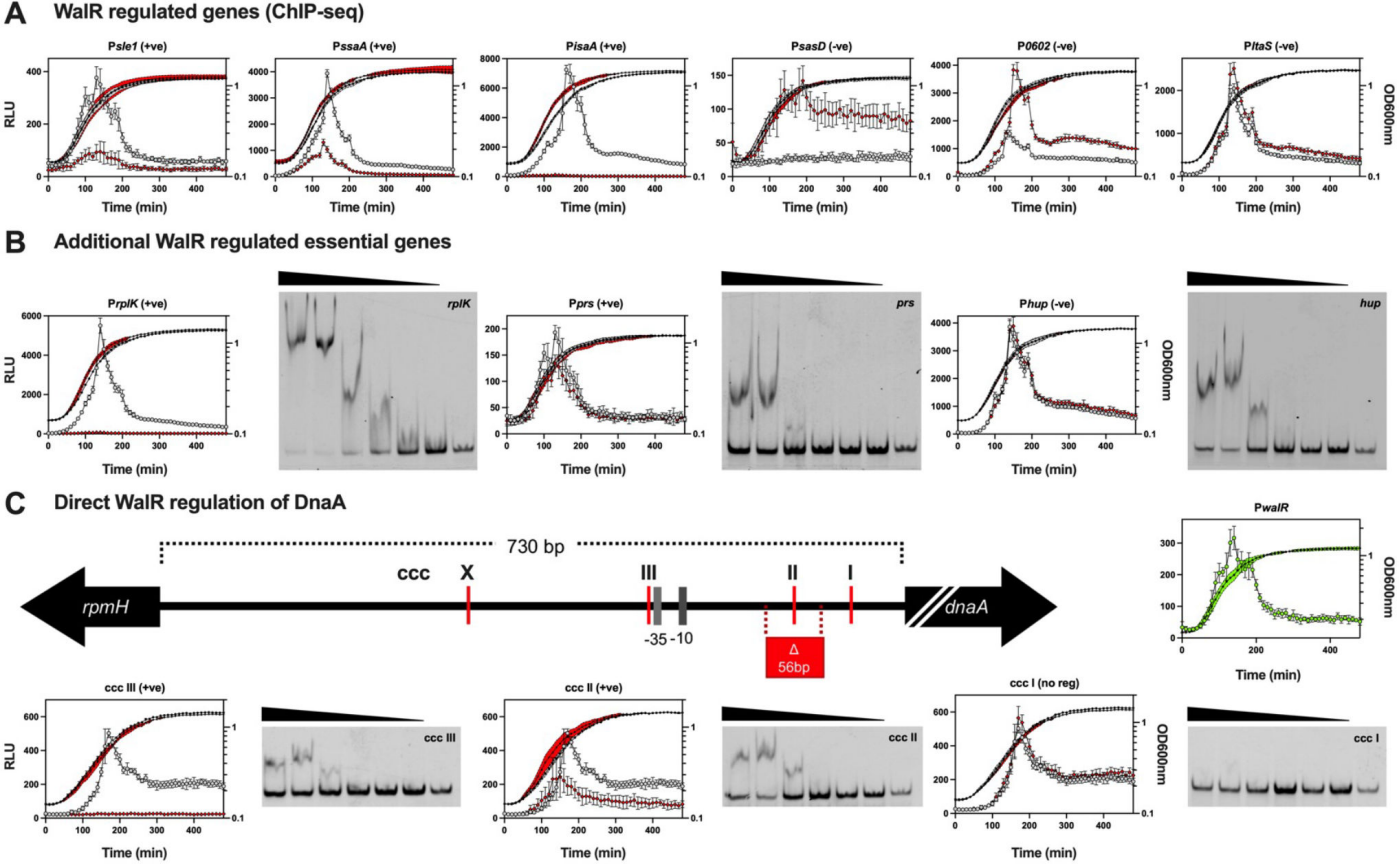
Temporal control of WalR on essential *S. aureus* gene expression. (**A**) Six ChIP-seq regulated (including the essential *ltaS*) and (**B**) additional essential regulated genes were coupled to bacterial luciferase reporters showing the changes in promoter activity of the Wt (open gray circle: RLU; filled black circles: OD_600nm_) or the ccc (open red diamond: RLU; filled black diamond: OD_600nm_) mutated WalR-binding site in LB media over time. Data represent the mean of three independent experiments (±standard deviation). Representative EMSA gels for WalR binding to the WalR motif in the essential genes are shown. A total of six doubling dilutions (plus a no protein control—right lane) of WalR starting at 20 µM. WalR expression is represented by green open circles (RLU), with growth indicated by filled black circles (OD_600nm_). (**C**) Schematic (to scale, except *dnaA*—truncation indicated by white back slashes) of the dnaA promoter region with putative WalR-binding sites is denoted. The Wt and the three *dnaA* motif mutants (ccc I-III) were analyzed as described above. Representative EMSA gels for WalR binding each WalR motif (ccc I-III) in the *dnaA* promoter region. EMSA were conducted as described above. Highlighted in red is the 56-bp deletion ^47^ and the −35 and −10 sites for the *dnaA* promoter. Site ‘X’ was not investigated.

To further characterize the seven essential genes within the predicted direct WalR regulon and *dnaD* (44, 45) (Table S3), we built a time-resolved picture of their expression. The promoter regions for these genes, with or without an abrogating mutation (TGT to CCC) in the WalR-binding site, were introduced into a luciferase reporter plasmid and transformed into *S. aureus* NRS384 (Fig. 7A through C). Expression from the *walR* promoter rapidly peaked in mid-exponential phase and then tapered, as has been observed previously (Fig. 7B) (46). Mutation of the WalR-binding site reduced luciferase expression for the positively regulated genes (*sle1*, *ssaA,* and *isaA*) and increased the expression for the negatively regulated genes (*sasD*, SAUSA300_0602, and *ltaS*), corroborating the fluorescence data (Fig. 7A). Loss of WalR-negative regulation was shown to relieve repression of *sasD*, while the impact on SAUSA300_0602 was most pronounced into the stationary phase of growth. Only a subtle difference in expression was observed for *ltaS*; mutational abrogation of WalR binding prevented “turning off” of gene expression, resulting in prolonged expression into the stationary phase (Fig. 7B). No regulation was detected for *tagG*, *tarF*, or *dnaD* (Fig. S8). However, the very low level of *dnaD* and *tarF* promoter activity under the conditions tested precluded a definitive determination. Whereas strong, positive WalR regulation was observed for *rplK* and to a lesser extent, *prs* (Fig. 7B). The *hup* gene was negatively regulated by WalR, with a small reduction in expression upon the transition into the stationary phase, similar to *ltaS*. For *hup*, there was a bi-phasic change at 190 min present in the Wt strain, which was lost when the binding site was mutated (Fig. 7B). For *rplK*, *prs*, and *hup*, binding to their respective WalR motifs was confirmed by EMSA (Fig. 7B).

Closer inspection of the large (730 bp) upstream intergenic region between the divergent *dnaA* and *rpmH* genes revealed multiple potential WalR-binding motifs upstream of *dnaA* (Fig. 6B). The three sites proximal to the *dnaA* gene were chosen for further analysis (“site X” was not investigated, (Fig. 7C). Sites ccc II and ccc III impacted the expression of *dnaA* while no change in expression was observed for the mutation of ccc I (Fig. 7C). We observed a decrease in expression for ccc II and complete abrogation of expression for ccc III, suggesting layered tuning of *dnaA* expression by WalR. The binding of WalR to ccc III and ccc II was validated by EMSA, while in line with the absence of regulation upon mutation, no WalR binding to ccc I was observed (Fig. 7C). Recently, a suppressor mutant with a deletion in the *dnaA* promoter that reduced the level of DnaA activity (initiation of DNA replication) was identified in a *S. aureus Δnoc* strain (47). This deletion removed a 56-bp region surrounding the WalR-binding site denoted by ccc II (Fig. 7C). In agreement with this previous observation, we identified reduced expression from the *dnaA* promoter upon abrogation of the ccc II WalR-binding site.

### Modulation of WalKR activity alters lipoteichoic acid structure

**Fig 8:**
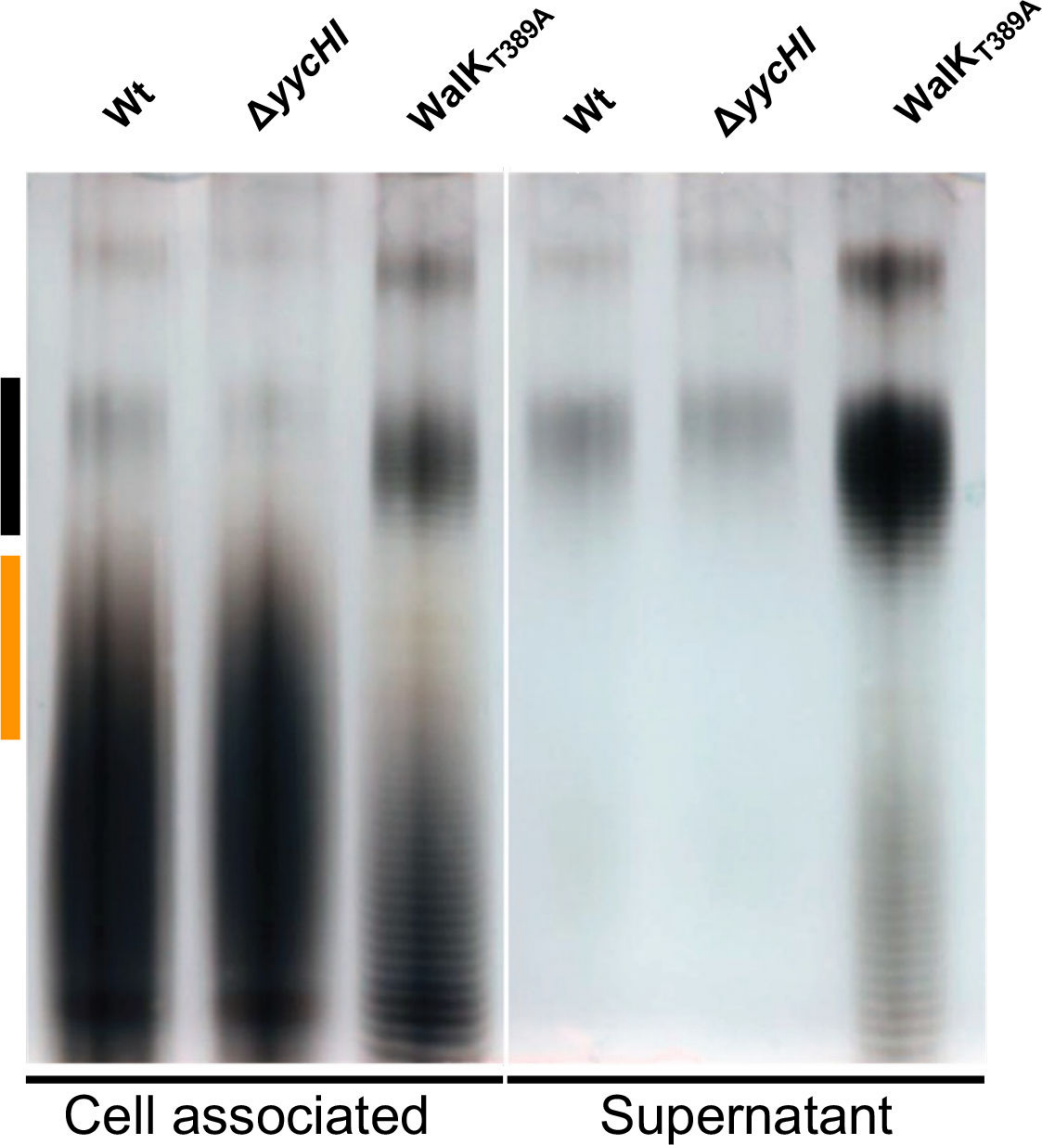
Assessing the influence of WalKR activity on LTA production. Polyacrylamide gel electrophoresis (PAGE) of purified LTA from NRS384 and isogenic mutant-derivative strains grown in LB. Each band indicates a glycerol phosphate polymer length of *n* + 1. Where bands are not clearly visible, this is due to heterogenous side chain modifications resulting in smearing patterns. Black and orange lines are shown and represent regions of distinct difference in LTA banding patterns between the strains.

Here, the physiological impact of WalR negative regulation of *ltaS* was assessed by extracting LTA (48) from our *S. aureus* mutants with contrasting WalKR activity. The Wt or a “down” mutant yielded a characteristic smeared banding pattern, indicative of heterogenous side-chain modification (49), whereas in a WalKR “up” mutant (WalK_T389A_), LTA chain length changes were observed. This manifested as a reduction in mid-length LTA (Fig. 8, orange line) and increase in high-molecular weight LTA (Fig. 8, black line). Analysis of the supernatant of the “up” strain resulted in LTA shedding, consistent with the compromised cell wall, characterized by sensitivity to lysostaphin (Fig. 1C).

### WalR controls DNA compaction by regulation of the DNA-binding protein HU

**Fig 9:**
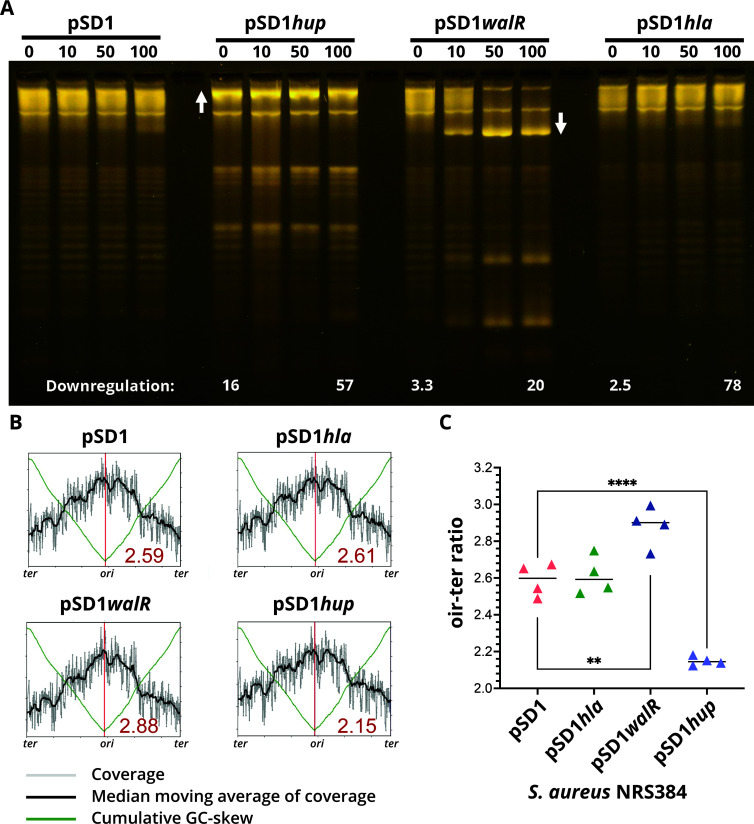
Impact of CRISPRi-mediated downregulation of *hup* or *walR* on DNA topology. (**A**) NRS384 containing either pSD1 (empty vector), pSD1*hup*, pSD1*walR*, or the negative control pSD1*hla* were induced with increasing concentrations of aTc (0, 10, 50, and 100 ng/mL). At 5 h post induction, total RNA and plasmids were isolated. Plasmids were run on a 1% (wt/vol) TBE gel containing 2.5 µg/mL chloroquine to allow for the resolution and visualization of discrete topoisomers of supercoiled plasmid DNA. Relative fold downregulation of each targeted gene compared with uninduced vector control (pSD1, 0) as determined by RT-qPCR is denoted at the bottom of the image. The image is representative of three repeat experiments. (**B**) A representative whole-genome sequencing read-coverage graph showing the ori-ter ratios for the four CRISPRi constructs under 100 ng/mL^−1^ aTc induction. Each plot represents one of four biological sequencing replicates for each CRISPRi construct. Shown in the bottom-right quadrant of each graph is the mean ori-ter ratio across the four replicates. The ori-ter ratio between the sequence read coverage at the origin and terminus. (**C**) Summary graph of ori-ter ratios for each of the four CRISPRi constructs induced with 100 ng/mL of aTc to downregulate expression of the three targeted genes (*hla*, *walR*, and *hup*). Shown are the mean of quadruplicate, independent biological replicate sequencing experiments. Differences between means assessed using unpaired Student’s *t*-test, **P = 0.0059 and ***P ≤ 0.0001.

The essential gene *hup* (50), which encodes the sole *S. aureus* DNA-binding protein HU, was shown here to be negatively regulated by WalR (Fig. 7B). *S. aureus* HU belongs to a family of low-molecular weight nucleoid-associated proteins (NAPs), and although it is largely uncharacterized, its structure has been determined (51). Orthologs of HU NAPs from other bacteria have previously been shown to control DNA compaction, introducing negative supercoils into relaxed DNA (52, 53), and play an essential role in the initiation of DNA replication in *B. subtilis* (54). To investigate potential changes in DNA topology mediated by *S. aureus* HU and WalR regulation of HU, we performed CRISPRi knockdown of *walR*, *hup*, and as a negative control, *hla*. The CRISPRi knockdown titrated the expression of each targeted gene (Fig. 9A), resulting in 57-, 20-, and 78-fold downregulation of *hup*, *walR*, and *hla*, respectively. There was some knockdown observed in the absence of an inducer, attributable to the leaky expression of the CRISPRi guide and the location of the guide (overlapping the promoter for *hup*). HU knockdown resulted in a relaxation of plasmid supercoiling, shown by increased DNA band intensity compared with control (Fig. 9A, white up arrow). Knockdown of *walR* had the opposite effect, indicated by increased DNA band intensities arising from faster migrating topoisomers with greater supercoiling density (Fig. 9A, white down arrow). Knockdown of *hla* and empty vector had no impact on DNA topology (Fig. 9A). These results are consistent with WalR-negative regulation of *hup* and show that *S. aureus* HU, as observed in other bacteria, increases the supercoiling density of DNA. The negative regulation of *hup* by WalR causes the relaxation of supercoiling and likely leads to decreased DNA compaction .

### Changes in initiation of DNA replication mediated by WalR and HU

We next assessed whether WalKR modulation of *hup* could also impact the initiation of DNA replication in *S. aureus*, as seen in *B. subtilis* by depletion of the orthologous protein (54). We used DNA sequencing to assess differences in the *ori*-to-*ter* ratios of the *S. aureus* CRISPRi knockdown constructs for *walR*, *hup*, *hla*, and vector control and aTc induction (0 or 100 ng/mL^−1^) (47, 54). We saw a significant reduction in ratios from 2.60 in the vector control and non-target *hla* control compared with 2.15 when the expression of *hup* was repressed (Fig. 9B and C), *P* < 0.0001]. A significant increase (2.88, *P* = 0.0059) in the mean *ori*-to-*ter* ratio was observed when *walR* was repressed (Fig. 9B and C), consistent with the negative regulation of *hup* by WalR (Fig. 7B). These data suggest that *S. aureus* HU contributes to promoting initiation of DNA replication and is directly influenced by WalKR-mediated regulation.

Collectively, our findings link diverse and crucial cellular processes, including DNA replication and cell wall homeostasis, to the activity of WalKR. Distinct from other Bacillota, WalKR appears to serve as a nexus for both regulatory and temporal coordination of these diverse activities.

## DISCUSSION

**Fig 10:**
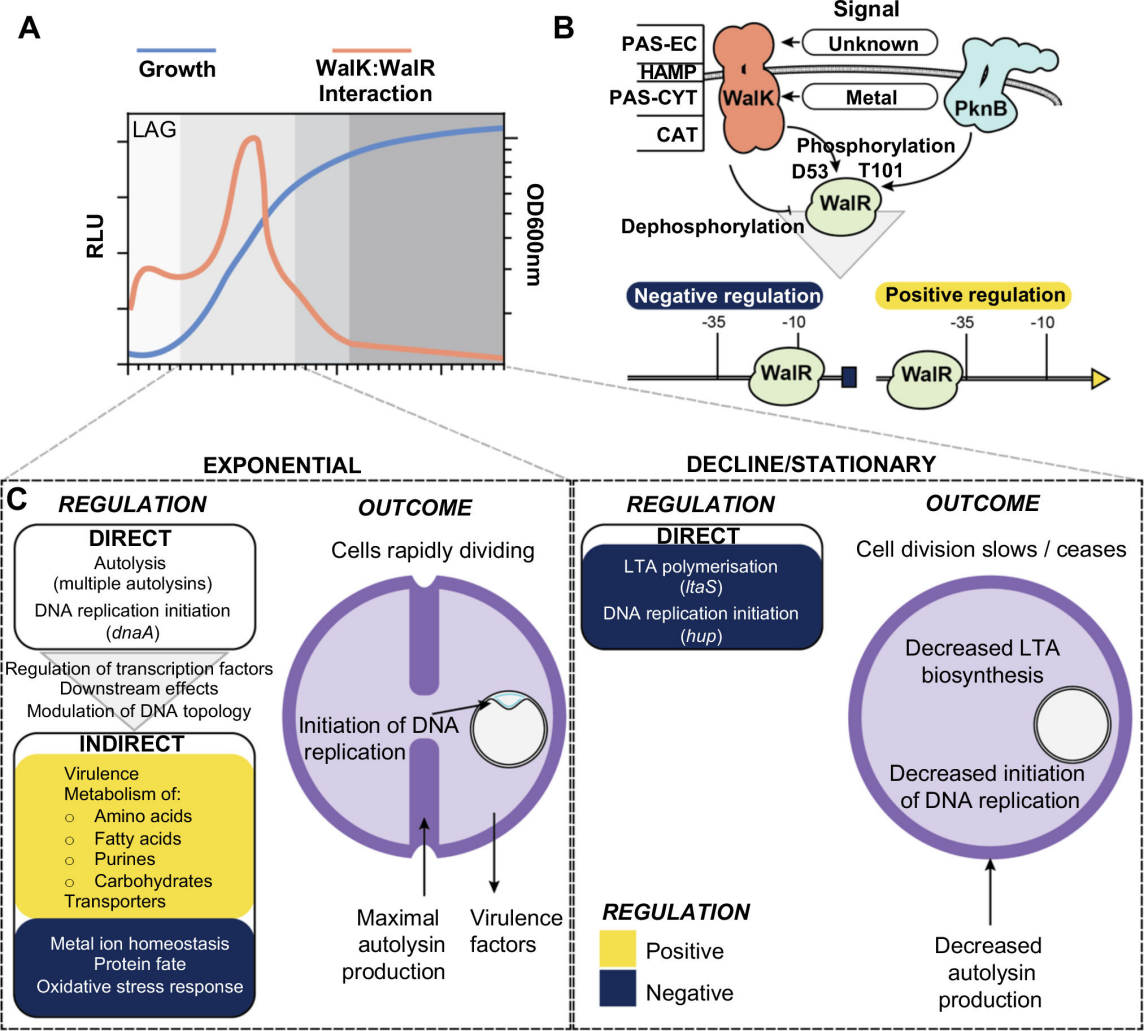
A model of the role of WalKR in cell division. (**A**) Changes in the interaction of WalK with WalR throughout growth. (**B**) Mechanism of signal transduction and transcriptional control of WalKR. −35 and −10 denote promoter regions. PAS-EC, Per-Arnt-Sim extracellular; HAMP, present in histidine kinases; PAS-CYT, PAS-cytoplasmic; CAT, catalytic domain. (**C**) Transcriptional changes and their outcomes for the direct and indirect WalKR regulon during different growth phases. Yellow and dark-blue denote positively and negatively regulated gene sets, respectively.

The reason for WalKR essentiality differs across the Bacillota (55). In *S. aureus*, WalKR essentiality is proposed to result from polygenic control of non-essential autolysins involved in the cleavage and relaxation of peptidoglycan crosslinks, allowing expansion of the cell wall (6). Here, we extend this understanding, showing WalKR direct control of at least five essential genes (*rplK*, *hup*, *ltaS*, *prs*, and *dnaA*) not directly involved in peptidoglycan biosynthesis but intimately linked with cell growth and cell division. Thus, we propose that WalKR essentiality arises though polygenic coordination of multiple cellular processes: ribosome assembly, peptidoglycan homeostasis, LTA polymerization, nucleotide metabolism, DNA topology, and the initiation of DNA replication, ultimately making WalKR an indispensable link between cell wall homeostasis and DNA replication. We propose a model in which WalK senses a currently unknown ligand during logarithmic growth through the extra-cytoplasmic PAS domain, resulting in autophosphorylation, dimerization, and maximal interaction between WalK and WalR (Fig. 10A). The level of WalK activation can also be dynamically tuned in response to the metalation state of the intracellular PAS domain (27). WalK:WalR interaction allows phosphotransfer to WalR residue D53, while a second WalR site T101 can be phosphorylated through the PknB kinase [recognizes muropeptide fragments (56)]. Phosphorylated WalR binds the cognate recognition motifs of its direct regulon as a dimer, causing either negative or positive changes to gene expression, primarily dependent on the position of the binding motif in relation to the transcriptional start site (Fig. 10B). The direct WalR regulon has three broad functions: (i) control of cell wall metabolism through regulation of a suite of autolysins governing peptidoglycan homeostasis and fine tuning of LTA biosynthesis through negative regulation of lipoteichoic acid synthase, (ii) linking initiation of DNA replication to cell wall homeostasis through regulation of *dnaA* and *hup*, and (iii) signal amplification through modulation of transcription factors, selection of other TCSs, and via negative regulation of DNA-binding protein HU (Fig. 7B). Together with the downstream effects of changes in cell wall metabolism, signal amplification drives changes in expression of the indirect WalKR regulon, producing a large shift in cellular transcription that includes the increased expression of virulence factors and metabolic genes (Fig. 10C).

We found that WalKR negatively regulates the expression of *ltaS*, during late exponential/stationary phase. To our knowledge, this is the first report of direct transcriptional regulation of *ltaS*, although post-translational regulation has been described (57). We also detected VraR binding to a site further upstream of the WalR site in the *ltaS* promoter. Though comparative transcriptomics, *ltaS* (N315 locus_tag SA0674) has previously been mapped to the VraSR regulon as a positive regulator (58); however, direct control was not demonstrated (38). It is not surprising that WalKR and VraSR regulate *ltaS* transcription as both TCSs are intimately connected to cell wall homeostasis, with VraSR governing the cell wall stress stimulon (59, 60). Together, LTA and wall teichoic acid are present in the Gram-positive cell wall in roughly equal proportion to peptidoglycan (61). LTA chain length is an intrinsic property of the LtaS enzyme that is dictated by the availability of lipid starter units (62); it is unlikely that the chain length differences observed upon activation of WalK are solely attributable to direct negative regulation of *ltaS*. In *S. aureus*, LTA modifications are performed by the *dltABCD* operon (D-alanylation) (63) and *yfhO*, *gtcA*, and *csbB* (glycosylation) (64), none of which were significantly changed upon WalK activation. We speculate that the global transcriptional rewiring of the cell upon WalK activation may affect the availability of LTA starter units for chain formation and modification, although this remains to be investigated.

We also found that WalKR controls essential genes involved in the initiation of DNA replication. In *S. aureus*, DNA replication is initiated by binding of DnaA to AT-rich regions at the origin of replication, *oriC* (65). This process is tightly controlled, as mistiming of initiation results in aberrant cell division (66). We showed that WalKR can both positively regulate the expression of *dnaA* and negatively regulate *hup*. The role of *hup* in staphylococcal DNA replication initiation has not previously been investigated, but in *B. subtilis*, the *hup* homologue *hbs* has been shown to promote initiation of chromosome replication (54). We show this function is conserved in *S. aureus*, as reduced expression of *hup* reduced initiation of DNA replication. In addition to *hup*, two other regulators of DNA replication initiation have been characterized in *S. aureus:* Noc and CcrZ (66). Noc is a negative regulator of DnaA-driven initiation (47), whereas CcrZ is a positive regulator (66). The mechanisms underlying the control of DnaA by these proteins are yet to be fully defined; however, it is unlikely that Noc directly regulates *dnaA* expression (47) and CcrZ may act post-translationally, by phosphorylating an unknown intermediate factor (66). A recent investigation into the role of Noc in *S. aureus* recovered a suppressor mutant that downregulated the activity of DnaA with a deletion of a 56-bp region (Fig. 7C) within the 5′UTR of *dnaA* (47). As this region encompasses one of two characterized WalR-binding motifs upstream of *dnaA*, we propose that the loss of positive WalR control through deletion of the binding site explains the observed decease in DnaA activity. Additionally, our RNA-seq showed an overall increase in *dnaA* expression (1.05 Log_2_FC - WalK_T389A_ vs Wt) in exponential phase upon WalK activation, further highlighting the positive role WalR has on *dnaA* expression.

That WalR was found to positively regulate *dnaA* and negatively regulate *hup*, both of which are promoters of initiation, is somewhat counterintuitive. Knockdown of *walR* expression caused over-initiation of chromosome replication, consistent with its negative control of *hup* but contradictory to its positive regulation of *dnaA* (Fig. 8B and C). It may be that loss of HU is a dominant phenotype, reducing initiation even in the presence of higher levels of DnaA or, alternatively, that regulation of each gene is temporally distinct. The latter hypothesis is supported by our observation that *dnaA* regulation occurred during exponential phase whereas regulation of *hup* was in the stationary phase (Fig. 7C). Exactly how WalKR transcriptional regulation of both *dnaA* and *hup* works in concert with post-translational control mediated by HU, Noc, and CcrZ remains to be elucidated. It is of note that during the discovery of WalR in *B. subtilis* using a temperature-sensitive mutant, anucleated cells were observed at the non-permissive temperature. This observation hints at the possibility of a wider association between WalR and DNA replication in other Bacillota (9).

HU is a multifunctional protein and contributes to DNA compaction, introduces negative supercoils into DNA (52), and can impact localized gene regulation (67). As we observed opposite changes to DNA supercoiling caused by depletion of *walR* and *hup*, we propose that the negative regulation of HU by WalR causes relaxation of supercoiling and may lead to decreased compaction of cellular DNA. Transcriptional regulation of *hup* expression has not previously been described in *S. aureus*. However, qualitative western blots have shown HU to be continuously present in the staphylococcal nucleoid throughout all growth phases (68). Taking these factors into account, we propose that WalKR regulation of *hup* is not a binary switch but rather provides tunable control of this essential system. Intriguingly, in *Mycobacterium tuberculosis*, the serine/threonine kinase PknB negatively regulates HU DNA binding through phosphorylation (69), and in *S. aureus*, PknB-mediated phosphorylation activates WalR (70). Furthermore, a PknB phosphorylation site on HU has been experimentally identified in *S. aureus* (71). Therefore, PknB may directly repress HU DNA binding through post-translational modification and indirectly represses the expression of *hup* through activation of WalR.

WalKR has long been considered a promising target for the development of novel anti-Gram-positive agents (72), although to date, no WalKR-targeted compounds have been successfully developed. Here, we show the system remains a viable target for conventional antibacterial chemotherapy due to its role as a signal-integrating nexus of essential cellular functions and the presence of two PAS domains capable of binding small molecules inside and outside of the cell (19, 27). Increasingly, alternative strategies to traditional antibacterial chemotherapy are being explored, including the development of the so-called “antibiotic resistance breaking (ARB) compounds” that re-sensitize resistant strains to existing antibiotics (73). We found that mutationally activated WalKR “up” mutants were more sensitive to three different antibiotic classes targeting the cell wall: oxacillin, tunicamycin, and vancomycin. For instance, mutation-induced stimulation of WalKR activity returned NRS384 MRSA to oxacillin sensitivity despite the presence of *mecA*, opening an alternative avenue for future WalKR- focused drug development; the discovery of ARB compounds that phenocopy WalKR activating mutations.

## MATERIALS AND METHODS

### Strains, oligonucleotides, media, and reagents

Bacterial strains and plasmids are listed in Table S5. Oligonucleotides (IDT) used in this study are listed in Table S6. *Escherichia coli* were routinely cultured in LB (Merck) or on L-agar [1.5% (wt/vol) agar added] unless stated otherwise. *S. aureus* were routinely grown on Brain Heart Infusion (BHI) Agar (Bacto, BD Biosciences) or Sheep Blood Agar. When cultured in broth, they were grown in Brain Heart Infusion Trypticase Soy Broth (TSB, Oxoid) or LB with shaking at 200 rpm. For selection, antibiotics (Sigma) were added at the following concentrations for *E. coli* (*E.c.*) and *S. aureus* (*S.a*.): ampicillin 100 µg/mL^−1^ – *E.c*., kanamycin 50 µg/mL^−1^ – *E.c*./*S.a*., and chloramphenicol 10 µg/mL^-1^ – *E.c*./*S.a*. Restriction enzymes, Phusion DNA Polymerase, and T4 ligase were purchased from New England Biolabs. Phire Hotstart II DNA polymerase for colony PCR was purchased from Thermo Fisher.

### *S. aureus* site-directed mutagenesis by allelic exchange

Upstream and downstream regions of the point mutation for *walK*_T389A_ (IM7/IM120/IM121/IM10), *walR*_T101A_ (IM31/IM232/IM233/IM10), or *spa* deletion (IMT275/IMT353/IMT354/IMT278) were PCR amplified, and then, a spliced overlap extension-PCR (SOE-PCR) was performed on the gel-extracted template to generate an amplicon for SLiCE cloning into pIMAY-Z (74). This yielded plasmids pIMAY-Z *walK*_T389A_, pIMAY-Z *walR*_T101A_, and pIMAY-Z Δ*spa*. To construct pIMAY-Z *walK*_Y32C_, genomic DNA from a sectored mutant of NRS384 Δ*yycHI* (containing an additional WalK_Y32C_ mutation) was amplified with primers IM107/IM10 and the amplicon cloned as described above. Construction of isogenic mutants of NRS384 by allelic exchange was performed as described previously (74). The WalK-enhancing mutations could visually be discriminated from the wild type due to reduced colony size and opacity. For the WalR_T101A_, the mutation was screened by colony PCR (70°C annealing temperature) with primers IM233/IM181. From putative mutants, genomic DNA was extracted from 1  mL of overnight culture (DNeasy Blood and Tissue Kit—Qiagen) pre-treated with 100 µg of lysostaphin (Sigma cat. no. L7386) and sequenced on an Illumina NextSeq by the Doherty Applied Microbial Genomics Facility (University of Melbourne). Resultant reads were mapped to a NRS384 reference genome (75) and mutations identified using Snippy (https://github.com/tseemann/snippy).

### Construction of pRAB11-FT

To construct a vector for the C-terminal FLAG tagging of *S. aureus* proteins, the anhydrotetracycline-inducible vector pRAB11 (34) was digested with KpnI to linearize and gel extracted. The 6.4-kb vector was then amplified with primers IM512/IM513 to add in a consensus ribosome-binding site (IM512), a 1xFLAG tag, and downstream *tonB* transcriptional terminator (IM513). The amplimer was digested with KpnI, gel extracted, re-ligated to yield pRAB11-FT. The sequence of the plasmid was verified by sequencing on the Illumina platform. To clone into pRAB11-FT, the vector was digested with KpnI, gel extracted, and used as template with primers IM514/IM515 to amplify the vector backbone. Response regulators (WalR—IM516/IM517), (SaeR—IM518/IM519), (VraR—IM520/IM521), and (HptR—IM522/IM523) were amplified from the start codon and omitting the stop codon with NRS384 genomic DNA. The products were gel extracted, SLiCE cloned into amplified pRAB11-FT, and transformed into IM08B, yielding pRAB11:*walR*^FLAG^, pRAB11:*saeR*^FLAG^, pRAB11:*vraR*^FLAG^, and pRAB11:*hptR*^FLAG^. The plasmids were electroporated into NRS384Δ*spa*.

### Construction of pIMC8-YFP reporter strains and assay for YFP activity

Promoter regions for *sasD* (IM1127/IM1107), *sle1* (IM1108/IM1109), *P602* (IM1129/IM1110), *ltaS* (IM1111/IM1112), *ssaA* (IM1130/IM366), and *isaA* (IM1128/IM364) were PCR amplified from NRS384 genomic DNA and gel extracted. The vector pIMC8-YFP (27) was digested with KpnI, gel extracted, and PCR amplified with IM1/IM385. The amplified promoters and vector were SLiCE cloned, transformed into IM08B, and subsequently electroporated into NRS384, NRS384 Δ*yycHI*, or NRS384 *walK*_Y32C_. Mutations disrupting the WalR motif (1st TGT to CCC) were introduced by SOE-PCR with the bracketed primers sets for *hpt* (IMT300/IMT301), *sle1* (IM1108/IM1115; IM1114/1109), *P0602* (IM1129/1119; IM1118/IM1110), *ltaS* (IM1111/IM1117; IM1116/IM1112), *ssaA*^CCCI^ (LS371/LS376; LS375/LS372), *ssaA*^CCCII^ (LS371/IM1121; IM1120/LS372), *isaA*^CCCI^ (IM1128/IM1123; IM1122/IM364), and *isaA*^CCCII^ (IM1128/IM1125; IM1124/IM364). The resultant mutated promoters were gel extracted, SLiCE cloned, transformed into IM08B, and subsequently electroporated into NRS384. For the *sasD* promoter, the mutation was incorporated into the reverse primer (IM1113) in combination with IM1127. To assess YFP production, each strain was grown in 5 mL of LB containing 10 µg/mL^−1^ chloramphenicol in a 50-mL tube for overnight at 37°C with shaking at 200 rpm. The culture was diluted 1:100 in 5 mL of fresh LB containing chloramphenicol and incubated overnight. The fluorescence (excitation 512 nm, emission 527 nm) of each strain (200 µL—Nunc black well plates) was read in triplicate using an Ensight Multimode Plate Reader (PerkinElmer) set to 100 flashes per well. Resultant data were plotted using the GraphPad Prism (v9.3.1) software package.

### Production and purification of proteins

For production of WalR, Rosetta 2 (DE3) pET28(a):*walR* was grown in 2 L of autoinduction media (76) at 25°C for 4 days with vigorous shaking, and cells were harvested by centrifugation. For production of VraR, Rosetta 2 (DE3) pET21(d): *vraR* was grown in 2 L of LB at 37°C with vigorous shaking to an OD600 nm of 0.6, chilled on ice for 10 min, and then induced with 1 mM IPTG. Subsequently, cells were grown for a further 16–20 h at 18°C with vigorous shaking and harvested by centrifugation. For purification of both proteins, cell pellets were resuspended at 3 mL/g in buffer A [50 mM NaH_2_PO_4_ (pH 8.0)] with 300 mM NaCl and 10 mM imidazole. To enhance lysis and prevent proteolysis, 7,000 U chicken egg-white lysozyme (Sigma), 2 complete EDTA-free protease inhibitor tablets (Roche), and 20 U DNase I (NEB) were added. Cells were lysed by sonication, and lysates were clarified by centrifugation at 30,000 × *g* for 30 min at 4°C. The cleared lysate was loaded onto a 25-mL free-flow gravity column (GeneFlow) packed with 3-mL TALON Metal Affinity Resin (Takara Bio) and washed with 10 column volumes (CV) of buffer A containing 20 mM imidazole and 2 M NaCl. The protein was eluted in 2 CV of buffer A containing 150 mM imidazole and 300 mM NaCl and dialyzed overnight with 6 kDa MWCO (CelluSep) into storage buffer [25  mM Tris (pH 8.0), 300  mM NaCl, 20  mM KCl, 10  mM MgCl_2_, 1  mM DTT, 5% glycerol]. The proteins were spin concentrated to 5 mg/mL (3 kDa MWCO, vivaspin 20, Sartorius), aliquoted, snap frozen in liquid nitrogen, and stored at −80°C until required.

### Electrophoretic mobility shift assay

DNA duplex probes for electrophoretic mobility shift assay binding assays were generated by annealing two 70-bp single-stranded oligonucleotides, one of which was labeled with Cy5 fluorophore on the 5′ end (Table S6). Annealing was performed in duplex buffer [30 mM HEPES (pH 7.5), 100 mM potassium acetate] by heating equimolar concentrations of complimentary single-stranded oligonucleotide at 94°C for 2 min and then cooling to room temperature over 30 min. Duplexes were subsequently gel extracted from a 2% (wt/vol) agarose 1×TBE gel. Binding reactions were performed in a final volume of 25 µL of binding buffer [25  mM Tris (pH 8.0), 300  mM NaCl, 20  mM KCl, 10  mM MgCl_2_, 1  mM DTT, 4 µg BSA, 0.5 µg salmon sperm DNA]. Initially, varying concentrations of WalR/VraR were incubated for 5 min at 25°C in binding buffer, then DNA probe was added to a final concentration of 16 nM, and the reaction was incubated for a further 15 min. After incubation, reactions were mixed (5:1) with 6× Orange G loading buffer (77) and 5 µL was electrophoresed on an 8% polyacrylamide native gel (29:1 acrylamide:bisacrylamide ratio) in 1×TBE at 4°C. Bands were visualized using a GE Amersham 600 imager in the Cy5 channel with a 10 min exposure time.

### Total RNA extraction and rRNA depletion

A 10-mL LB (50-mL tube) culture was grown overnight at 37°C with shaking at 200 rpm. The saturated culture was diluted 1:100 into fresh 10-mL TSB and grown to an OD_600nm_ of 0.8–1.0. A 5-mL aliquot of culture was removed and added to 10 mL of RNAprotect Bacteria Reagent (Qiagen) and mixed by vortexing. The sample was incubated at room temperature for 5 min. Cells were then harvested by centrifugation (7,000 *× g*/5 min/22°C), the supernatant was discarded, and the cell pellet was resuspended in 1 mL of TRIzol (Invitrogen). Cells were lysed by bead beating (Precellys 24 instrument—6000 rpm, 1 min, 100 µm zirconium beads), and cell lysates were clarified by centrifugation (20,000 *× g*/10 min/4°C). Subsequently, 700 µL of supernatant was removed and mixed with 700 µL of ethanol. RNA was extracted using a Direct-Zol RNA Miniprep Plus Kit (ZymoResearch) according to the manufacturer’s instructions, including the on-column DNase I treatment step. Following RNA extraction, an additional DNA removal step was performed using a TURBO DNA-Free Kit (Invitrogen) according to manufacturer’s instructions. The absence of DNA was accessed *by gyrB* PCR (IM1020/IM1021) on 1 µL of RNA template, yielding no amplification. RNA quality was determined on the Bioanalyser (Agilent) with all yielding an RNA integrity number of above 8. For each strain, three independent RNA extractions were made. A 5-µg aliquot of total RNA was depleted for rRNA with the mRNA then converted into cDNA with the ScriptSeq Complete Bacteria Kit (Epicentre). The libraries were sequenced on the Illumina HiSeq platform for 50-bp single end reads. RNA-seq reads can be found under Bioproject: PRJNA875030.

### RNA-seq data analysis

RNA-seq data were analyzed using the *S. aureus* USA300 FPR3757 reference genome (accession number: NC_007793) and Kallisto (78), a kmer-based pseudoalignment tool, with analysis and visualization using Degust (https://github.com/drpowell/degust). Degust uses Voom/Limma (79) and generates an interactive website to analyze and explore the data.

### Preparation of samples for ChIP-seq

The four plasmids and an empty vector control (*walR*^FLAG^ / *vraR*^FLAG^ / *hptR*^FLAG^ / *saeR*^FLAG^ / pRAB11^FLAG^) were each transformed into the CA-MRSA USA300 strain NRS384 with *spa*, which encodes Protein A, deleted to reduce non-specific IgG-binding during immunoprecipitation. An overnight culture (10-mL LB in a 50-mL tube) of each strain NRS384Δ*spa* containing either pRAB11: *walR*^FLAG^ / *vraR*^FLAG^ / *hptR*^FLAG^ / *saeR*^FLAG^ or pRAB11^FLAG^ only was grown at 37°C with shaking at 200 rpm. Overnight cultures were diluted 1:100 in fresh LB (100 mL) in a 1-L baffled flask and grown to an OD_600nm_ of 0.5, induced with 100 ng/mL^−1^ of anhydrotetracycline and grown for a further hour. Cells were crosslinked by direct addition of methanol-free formaldehyde (Pierce) to cultures [final concentration of 1% (vol/vol)] and incubated with gentle mixing (rotating platform) for 15 min at room temperature. The crosslinking reaction was quenched by the addition of glycine to a final concentration of 400 mM and incubated at a further 15 min. Cells were pelleted (7,000 × *g*/10 min/4°C) and washed three times with ice-cold phosphate-buffered saline (PBS), and the pellet was stored at −80°C. For cell lysis, cells were suspended in 1 mL of lysis buffer [50 mM Tris-HCl (pH 7.4), 150 mM NaCl, 0.1% Triton X-100, 100 µg/mL^−1^ lysostaphin (Ambi), 500 µg/mL^−1^ RNaseA, complete miniprotease inhibitor EDTA free containing 100 um zirconium beads] and incubated for 20 min at 37°C. The weakened cells were then disrupted using bead beating (6000 rpm for 1 min; Precellys 24 instrument), and the cell lysate was clarified by centrifugation (20,000 *× g*/10 min/4°C). The cation concentration of the lysate was adjusted with MgCl_2_ and CaCl_2_ to 100 mM. The DNA was sheared by the addition of a range of DNase I (NEB) concentrations (0.5–2U), in a 200 µL volume, followed by incubation at 37°C for 10 min. The reaction was quenched by the addition of EDTA to 50 mM on ice. The degree of DNA fragmentation was assessed by electrophoresis of samples on a 2% (vol/vol) agarose TAE gel. Samples showing maximal fragmentation at between 100 and 300 bp were subjected to immunoprecipitation. Lysis buffer (made up to 4 mL—omitting lysostaphin and RNase A) containing 10 µg of M2-anti FLAG antibody was added and incubated overnight at 4°C on a rotating platform in a 15-mL tube. A 100-µL aliquot of Protein G agarose (Pierce) was added to the lysate and incubated a further 2 h at room temperature. The sample was centrifuged (2,500 *× g*/3 min/22°C), and the agarose pellet was washed three times with 4 mL of IP buffer [25 mM Tris.Cl (pH 7.2), 150 mM NaCl] and finally resuspended in 200 µL of elution buffer [10 mM Tris.Cl (pH 8), 1 mM EDTA, 1% SDS containing 100 µg of proteinase K]. Crosslinks were reversed incubated for 2 h at 37°C and then 9 h at 65°C with shaking at 1,400 rpm. Finally, eluted DNA was cleaned up by PCR purification (QiaQuick PCR Purification Kit, Qiagen). DNA libraries were prepared with NEBNext Ultra II DNA Library Prep Kit for Illumina and sequenced using an Illumina MiSeq.

### ChIP-seq read mapping, peak identification, and motif searching

MiSeq reads from each of the five experiments (pRAB11: *walR*^FLAG^ / *vraR*^FLAG^ / *hptR*^FLAG^ / *saeR*^FLAG^ or pRAB11^FLAG^ only) were first mapped to the *S. aureus* USA300 NC_007793 reference chromosome using *samtools*. The resulting .*bam* and .*sam* files were used to create tag counts (i.e., mapped reads) with the *makeTagDirectory* script within *homer* (80). The *homer* peak identification tool (*findPeaks*) was extensively explored, but high levels of background reads were detected and precluded further use of *homer* for peak detection. An alternative strategy was developed by building read coverage plots for viewing in Artemis (81), using *samtools* and the command *% samtools depth -aa [target_file_name].bam [subtraction_file_name].bam | cut -f3,4 | perl -nae 'use List::Util qw(max); print max(0, $F[0]-$F[1]),"\n";' > [target_peaks].userplot*. This created a coverage plot of those regions of the *S. aureus* USA300 NC_007793 chromosome specifically bound by a given response regulator, relative the response regulator sequence reads in the subtraction set. These subtraction sets were a concatenation of a random selection of 20% of the sequence reads for the three response regulators and the plasmid-only control combined. Thus, for peak discovery of WalR-binding sites, an Artemis userplot was prepared from *vraR*^FLAG^ / *hptR*^FLAG^ / *saeR*^FLAG^ and pRAB11^FLAG^ subtracted from *walR*^FLAG^. The same method was used to generate userplots and discover binding peaks for the remaining three response regulators. The “*create feature from graph function*” in *Artemis* was then used to define chromosome regions represented by the subtraction coverage plots. These regions were mapped to the NRS384 genome in Geneious Prime (v2023.03) and searched using TGTNNNNNNNNTGT ±5 bp as input. Output sequences were combined with motif regions ±5 bp for the following previously experimentally validated WalR regulon members: SAUSA300_0739, SAUSA300_0955, SAUSA300_2051, SAUSA300_2253, SAUSA300_2503 (12, 24), and input to WebLogo (82). The resultant sequence logo was converted to a IUPAC code and used to search the NRS384 genome using Geneious Prime (v2023.03). ChIP-seq reads can be found under Bioproject: PRJNA875030.

### Mapping TSS in relation to WalR binding sites

The *S. aureus* NRS384 genome was annotated in Geneious Prime (v2023.03) with predicted transcriptional start sites as defined in a previous study (39). The 500-bp upstream of a predicted TSS were extracted and manually annotated with −35 and −10 elements and predicted WalR binding sites. *t*-tests (run in Stata v16.0) were used to test associations between WalR binding site position and orientation, with gene expression data from RNA-seq.

### Essentiality analysis

Essentiality of *S. aureus* genes was called if a locus had been described as essential in two previous studies (41, 42) and did not harbor a transposon insertion in the Nebraska transposon library (43). Genes were defined as belonging to the core genome if they were present in every strain of the *Aureo*Wiki orthologue table (https://aureowiki.med.uni-greifswald.de/download_orthologue_table) (83). Data from RNA-seq, ChIP-seq, and the essentiality and core genome analysis were integrated in R (v4.0.3, https://www.r-project.org/) with RStudio 2022.02.0+443 using dplyr (v1.0.8) and tibble (v3.1.6) and then visualized using UpSetR (v1.4.0) (43) with ggplot2(v3.3.5).

### Growth curves

*S. aureus* was grown overnight in LB at 37°C and subsequently diluted into fresh LB to an OD_600nm_ of 0.05. Growth was measured for 8 h at 37°C with 300 rpm dual-orbital shaking in a 96-well plate (Corning) using a Clariostar Plus (BMG) plate reader.

### Antibacterial susceptibility testing

Minimum inhibitory concentrations of antibacterial agents were determined by broth microdilution; bacteria were exposed to twofold serial dilutions of antibacterial agents in Mueller-Hinton broth 2 (BBL, BD) according to the guidelines provided by the Clinical and Laboratory Standards Institute. Vancomycin susceptibility was assessed using gradient plates as previously described (84).

### Lysostaphin sensitivity

An overnight 5-mL BHI culture of each strain was diluted 1:100 in an Eppendorf tube containing fresh BHI containing different final concentrations (0–1.6 µg/mL^−1^) of lysostaphin (Ambi). Cells were then incubated statically for 90 min at 37°C in a water bath with the CFU/mL^−1^ determined by dilution and spot plating onto BHI agar. Plates were incubated for 18 h at 37°C before enumeration.

### LTA extraction, purification, and analysis by PAGE

For extraction and analysis of LTA, 50 mL of LB was inoculated with a single colony of NRS384, NRS384 Δ*yycHI*, NRS384 *walK*_T389A_, and grown at 37°C with vigorous shaking for 18 h. Cells were harvested from 30 mL of saturated culture by centrifugation of (5,000 × *g*, 10 min) and LTA was extracted and analyzed by PAGE as described previously (49).

#### CRISPRi constructs and knockdown analysis

To generate pSD1 CRISPRi knockdown constructs for *walR*, *hla*, and *hup,* primers corresponding to the previously described guide RNAs for *walR* (IM1180/IM1181) and *hla* (IM1182/IM1183) were synthesized from Zhao *et al.* (85). Knockdown primers for *hup* [targeted the region overlapping and upstream of the *hup* start codon (IM1559/IM1560)] were designed with the annealed primer pairs for *walR*, *hla*, and *hup* cloned into the SapI site of pSD1 as described previously (86). Plasmids were then transformed into NRS384. Overnight cultures (5-mL LB, chloramphenicol 10 µg/mL^-1^ in 50-mL tubes) were then diluted in fresh media 1:100 (12.5ml LB) containing different concentrations of aTc to induce expression of dCAS9, with the optical density of the cultures followed.

For RNA isolation, RNA was isolated from 1 mL of cells (induced with either 0 or 100 ng/mL^−1^ of aTc) after 5 h of growth, as described above. A 1-µg aliquot of total RNA was converted into cDNA with Superscript IV and random hexamers as described previously (87). For RT-qPCR, 1 µL of cDNA was used as template with primers for *gyrB* (IM1020/IM1021), *hla* (IM1026/IM1027), *walR* (IM1153/IM1154), and *hup* (IM1586/IM1587) with Luna Universal qPCR Master Mix (NEB) on a QuantStudio 1 PCR Machine. The data were normalized to the *gyrB* gene and analyzed with the ΔΔ CT method (88).

For plasmid isolation, plasmid DNA was isolated from 10 mL of cells at 5 h post induction (induced with 0, 10, 50, and 100 ng/mL^−1^ aTc). Cells were centrifuged at 7,000 *× g*/2 min; pellet was washed in 1 mL of PBS and then resuspended in 400 µL of the resuspension buffer (Monarch Miniprep Kit—NEB) containing 50 µg of lysostaphin. The cells were lysed at 37°C for 30 min and then processed following the kit instructions through one column with elution in 30 µL of elution buffer. DNA was quantified with the Qubit BR DNA Quantification Kit and normalized to 15 ng/µL, with 150 ng of purified plasmid run on a 1% TAE gel. Subsequent 1% agarose gels (in 2×TBE) containing 2.5 µg/mL^−1^ chloroquine were run as described by Cameron *et al.* (89). Gels (10 cm) were run at 10 V for 16 h which were washed twice (30 min each wash) in dH_2_O and then stained with Sybr Gold for 30 min and subsequently imaged.

For genomic isolation, genomic DNA was isolated from the equivalent of OD_600nm_ of 5 after 5 h of growth (induced with 0 or 100 ng/mL aTc). The cell pellet was washed with 1 mL of PBS and resuspended in 90 µL of PBS containing 5 µL of 20 mg/mL^−1^ RNase A, 50 µg of lysostaphin, and 100 µL of the tissue lysis buffer (Monarch Genomic DNA Purification Kit, NEB). Cells were lysed at 37°C for 30 min and then processed following the manufacturer’s instructions.

### Analysis of *ori-ter* ratios

To measure to *ori-ter* ratios, genomic DNA prepared after CRISPRi knockdown (as above) was sequenced using the Illumina NextSeq (by the Doherty Applied Microbial Genomics Facility, University of Melbourne). Illumina reads were processed and analyzed using iRep, as previously described [v1.1 https://github.com/christophertbrown/iRep (90)].

#### Construction of pSmBIT and pLgBIT split luciferase vectors

TCS phosphoryl transfer from the sensor histidine kinase to the cognate DNA-binding response regulator requires direct interaction. To assess the impact of the “up” and “down” mutations on interaction dynamics between WalK and WalR across growth, we implemented a split luciferase system. Proteins were C-terminally tagged (separated by a glycine serine linker) with either the small bit (SmBIT—11 amino acids) or large bit (LgBIT—17.6 kDa) to reconstitute a functional luciferase that emits light in the presence of the furimazine substrate. Our modifications allowed the kinetics of protein-protein interaction to be non-invasively measured throughout *S. aureus* growth. The vector pRAB11(pC194 replicon) (34) was modified by PCR to restore the consensus *tetO* upstream of the *tetR* gene (IM1290/IM1291) (91). As described previously, this reduced the impact of elevated-level TetR production and allowed leaky expression of the target gene in the absence of aTc. The above 6.4-kb PCR product was gel extracted, treated with SLiCE, and transformed into *E. coli* IM08B, yielding pRAB11*. To introduce a consensus ribosome binding site and nine-nucleotide spacer before the start codon (AGGAGGAATTGGAAA) downstream of the two *tetO* sites (proceeding the gene of interest), pRAB11* was first digested with KpnI and gel extracted. This was used a template in a PCR (IM513/IM1355), and the product was digested with KpnI, gel extracted, and ligated. The ligation product was transformed into IM08B yielding pRAB11*RBS. The tetR*-RBS fragment was digested from pRAB11*RBS (SphI/KpnI) and ligated into complementary-digested pCN34 (pT181 replicon) (92) yielding pCN34*RBS. Both pRAB11*RBS and pCN34*RBS were digested with KpnI, gel extracted, and used as template in a PCR (IM515/IM1356). The following combinations were combined with 50 ng of each (i) pRAB11*RBS PCR and LINKER(GSSGGGGSGGGGSSG)-SmBIT gBlock and (ii) pCN34*RBS PCR and LINKER-LgBIT gBlock. gBlock sequences were codon optimized for *S. aureus.* The SLiCE reactions were transformed into IM08B yielding either pSmBIT or pLgBIT, with both vectors were fully sequenced to validate.

#### Cloning into split luciferase vectors

Either pSmBIT or pLgBIT was digested with KpnI, gel extracted, and used as template for PCR with primers IM515/IM1360. The pSmBIT or pLgBIT amplimers were combined with amplified open reading frames with stop codon removed and tailed with 5′-GATAGAGTATGATGAGGAGGAATTGGAAA-3′ forward or 5′-GAACCACCACCACCACTAGAACC-3′ sequences complementary to the vector.

WalR and WalK alleles were PCR amplified with IM1363/IM1364 and IM1365/IM1366, respectively, then SLiCE cloned into pSmBIT (for *walR* alleles) or pLgBIT (for *walk* alleles), and transformed into IM08B. For *S. aureus* transformations, at least 1 µg of pLgBIT(+*walK* allele) and pSmBIT(+*walR* allele) were purified from IM08B and co-electroporated into NRS384 with selection on BHI agar containing 10 µg/mL^−1^ chloramphenicol and 50 µg/mL^−1^ kanamycin.

#### Chromosomal tagging of WalR-SmBIT and WalK-LgBIT

To assess the functional interaction of WalR/WalK under native levels of protein of production, the native copy on the chromosome was tagged with SmBIT for WalR and LgBIT for WalK. The regions of DNA were assembled as follows: (i) *walR*-SmBIT-*walK: walR*-SmBIT was amplified from pSmBIT-*walR* with IM107/IM1517 and a downstream fragment encompassing *walK* was amplified with IM1516/IM10 from NRS384 genomic DNA. Both were gel extracted and joined by SOE-PCR. (ii) *walK*-LgBIT-*yycH: walK*-LgBIT was amplified from pLgBIT-*walK* with IM7/IM1519 and a downstream fragment encompassing 500 bp of *yycH* was amplified with IM1518/IM44 on NRS384 genomic DNA. Both were gel extracted and joined by SOE-PCR. Either amplimer was SLiCE cloned into pIMAY-Z and transformed into IM08B. The above cloning steps and *S. aureus* allelic exchange were performed as described by Monk and Stinear (74). The presence of *walR-*SmBIT and *walK-*LgBIT was screened by colony PCR with IM1360/IM1368 and IM1360/IM44, respectively. Genomic DNA was isolated from the strains and whole-genome sequenced.

#### Growth and luciferase curves

An overnight 5-mL LB containing antibiotics (in a 50-mL tube) were grown overnight. The culture was diluted 1:100 in fresh LB supplemented with chloramphenicol and kanamycin including a 1:5,000 dilution of the Nano-Glo Luciferase Assay Substrate (Promega). Preliminary growth curves in the presence of the substrate showed no impact on growth at this concentration. The culture was then dispensed in triplicate (200 µL) black/clear bottom 96-well plates (Cat no. 165305, ThermoFisher). The plates were sealed with MicroAmp Optical Adhesive Film (ThermoFisher) and incubated at 37°C with dual-orbital shaking at 300 rpm (Clariostar Plus, BMG). Every 10 min, the plate was read at OD_600nm_ and light emission (1 s exposure) collected over an 8-h period.

#### Bacterial luciferase reporter plasmid

To construct pIMK1-LUX, the Listeria phage integrase vector pIMK was digested with SphI/BglII to excise the PSA integrase and replace it with the PCR-amplified (IM1241/IM1242) low-copy number pSK41 replicon from pLOW, yielding pIMK1. Vectors pIMK1 and pPL2*lux* were then digested with SalI/PstI and the gel-extracted pIMK1 backbone ligated to the bacterial luciferase operon from pPL2*lux*. The vector pIMK1-LUX produces exact promoter fusions which can be cloned into the SalI/SwaI-digested vector, as described previously (93). Promoters for *walR* (IM1222/IM32), *sasD* [IM1216/IM248 (Wt) or IM1217(ccc)], *sle1* (IM1295/IM1115; IM1114/IM1296), *0602* (IM1294/IM1119; IM1118/IM1062), *ltaS* (IM1218/IM1117; IM1116/IM1219), *ssaA^CCCII^* (IM1297/IM1121; IM1120/IM1298), *isaA^CCCI^* (IM1220/IM1123; IM1122/IM1221), *isaA^CCCII^* (IM1220/IM1125; IM1124/IM1221), *tarF* (LS451/LS448; LS449/LS452), *tagG* (LS457/LS454; LS455/LS458), *dnaA* (IM1745/IM1744; IM1743/IM1746), *dnaD^CCCI^* (LS471/LS466; LS467/LS472), *dnaD^CCCII^* (LS471/LS468; LS469/LS472), *rplK* (IM1734/LS143; LS144/IM1735), *hup* (IM1289/IM1290; IM1291/IM1292), *dnaA^CCCI^* (LS443/LS440; LS441/IM1746), *dnaA^CCCII^* (LS443/LS438; LS439/IM1746), *dnaA^CCCIII^* (LS443/IM1743; IM1744/IM1746), and *prs* (IM1285/LS139; IM140/IM1288) were PCR amplified from genomic DNA with either the outer set (Wt promoter) or SOE-PCR with the four primers (ccc promoter). The amplimers were digested with SalI, gel extracted, and cloned into the above double-digested vector. Plasmids isolated from IM08B were transformed into NRS384 Wt. An overnight 5-mL LB culture with kanamycin was diluted 1:100 in fresh LB containing kanamycin with growth curves and light emission measured as described above for the split luciferase with the substrate omitted.

### Data visualization

Graphs were generated in R (v4.0.3, https://www.r-project.org/) or GraphPad Prism (v9.3.1) software packages.

## Data Availability

All data and links to data are available in the main text or the supplementary materials.

## SUPPLEMENTAL MATERIAL

Supplemental material mbio.02262-23-s0001.pdf Figures S1 to S9 and Tables S1 to S6.

ASM does not own the copyrights to Supplemental Material that may be linked to, or accessed through, an article. The authors have granted ASM a non-exclusive, world-wide license to publish the Supplemental Material files. Please contact the corresponding author directly for reuse.
